# NRF1 predominantly causes EZH2 overexpression in cancer cells

**DOI:** 10.1038/s41419-026-08861-4

**Published:** 2026-05-16

**Authors:** Juanli Qiao, Zhaojun Liu, Liankun Gu, Dajun Deng

**Affiliations:** Key Laboratory of Carcinogenesis and Translational Research (Ministry of Education/Beijing), Division of Etiology, Peking University Cancer Hospital and Institute, Fu-Cheng-Lu #52, 100142 Beijing, Haidian District China

**Keywords:** Targeted therapies, Translational research, Transcriptional regulatory elements

## Abstract

EZH2 is an oncogene and therapeutic target. Only a small proportion of cancer patients benefit from treatment with EZH2 inhibitors (EZH2is). The mechanisms underlying EZH2 overexpression and EZH2i resistance are not clear. Here, we report that the nuclear respiratory factor 1 gene (*NRF1*) is the gene whose expression is most strongly correlated with that of the *EZH2* gene in various cancer cell lines and that changes in *NRF1* expression consistently cause changes in *EZH2* expression in cancer cells. Mechanistically, as a transcription factor, NRF1 directly binds to the NRF1-binding sequence within the *EZH2* promoter and increases *EZH2* promoter activity. Deletion of the DNA-binding motif within the NRF1 or NRF1-binding sequence within the *EZH2* promoter abolishes the effects of NRF1 on *EZH2* expression. Notably, we further found that the status of NRF1 expression affected the sensitivity of human cancer cells to EZH2is, including GSK343 and tazemetostat. The sensitivity of cancer cells actively expressing both *NRF1* and *EZH2* to EZH2i is significantly greater than that of cancer cells actively expressing individual *EZH2* or *NRF1* alone and much greater than that of cancer cells expressing low levels of *EZH2* and *NRF1*. The effect of NRF1 on the sensitivity of cancer cells to EZH2is is EZH2 dependent. In conclusion, our findings reveal that NRF1 is a dominant cause of EZH2 overexpression in human cancers and that NRF1 overexpression increases the sensitivity of cancer cells to EZH2is. Active NRF1 and EZH2 expression may be a useful combined predictor for the treatment of cancers with EZH2is.

## Significance

We found that NRF1, as a transcription factor, predominantly drives EZH2 overexpression in cancers, and high NRF1 and EZH2 coexpression is a predictor of the sensitivity of cancer cells to EZH2 inhibitors.

## Introduction

The epigenetic modulator Enhancer of Zeste Homolog 2 (EZH2) is the core catalytic subunit of Polycomb Repressive Complex 2 (PRC2), which typically represses the transcription of target genes by catalyzing the trimethylation of lysine 27 of histone H3 (H3K27me3) in chromatin and activating target gene transcription via a noncanonical pathway [1]. Whereas EZH2 physiologically regulates a wide range of biological processes, including cell cycle progression, autophagy, apoptosis, DNA damage responses, cellular senescence, and cell fate decisions [2–5], it is also universally overexpressed in human cancers across many organs as a master determinant of cancer phenotypes [6–12]. EZH2 overexpression not only enhances cancer cell proliferation, migration, and invasion but also leads to poor prognosis in cancers [11, 12]. However, the mechanisms underlying EZH2 overexpression in cancer cells remain unclear.

As a cancer driver, EZH2 overexpression is a therapeutic target in the treatment of cancers. For example, the EZH2 inhibitors (EZH2is) tazemetostat and valemetostat have been approved for the treatment of non-Hodgkin’s lymphoma, rare adult sarcoma, and T-cell leukemia/lymphoma [13–16]. A number of other EZH2is, such as GSK343 [17] and GSK126 [18], are under development. EZH2is may inhibit the growth of cancer cells not only by blocking H3K27me3 modification but also by improving immunotherapy responses through reactivating antigenic retrovirus elements in the genome [17, 19, 20]. Unfortunately, EZH2i resistance is unavoidable [21, 22], and clinical biomarkers to predict the efficiency of EZH2is are in demand.

Nuclear respiratory factor 1, encoded by the *NRF1* gene (NCBI Gene: 4899), is a transcription factor that directly regulates several nuclear-encoded electron transport chain proteins [23]. NRF1 plays an essential role in mitochondrial biogenesis by coactivation with peroxisome proliferator-activated receptor gamma coactivator 1α (PGC-1α) [24]. NRF1 also controls the expression of genes that are associated with DNA replication, cell proliferation, and apoptosis [25]. Many studies have shown that NRF1 may participate in tumor progression. For example, NRF1 promotes spheroid survival and mesenchymal transition in breast cancer [26, 27]. However, the effects of the *NRF1* gene on cancer development and its mechanisms are largely unknown.

In accordance with the extensive coexpression status of the *NRF1* and *EZH2* genes in various cancer tissues and our pilot study [28], in the present study, we systemically studied the contribution of NRF1, a transcription factor, to EZH2 overexpression in cancer cells. Its effect on the sensitivity of cancer cells to EZH2is was also investigated in vitro and in vivo.

## Materials and methods

### Cell lines and cultures

The human HCT116 and SW480 colon cancer cell lines were kindly provided by Professor Yuanjia Chen at Peking Union Medical College Hospital. The RKO cell line was kindly provided by Professor Guoren Deng at the University of California. The cell lines LoVo, HEK293T, H1299, A549, and HepG2 were kindly provided by Professors Chengchao Shou, Zhiqian Zhang, and Qingyun Zhang at Peking University Cancer Hospital. The H460 cell line was purchased from the Cell Resource Center, Peking Union Medical College. The primary/early-passage normal human female colon fibroblast CCD-18Co cell line was purchased from American Type Culture Collection (CRL-1459, ATCC, Maryland, USA). These cells were grown in a 5% CO_2_ atmosphere at 37^°^C in RPMI 1640 or MEM medium (GIBCO, Carlsbad, USA) supplemented with 10% fetal bovine serum (GIBCO) and 1% penicillin‒streptomycin‒glutamine (GIBCO). These cell lines were tested and authenticated by Beijing Jianlian Genes Technology Co., Ltd. Short tandem repeat (STR) patterns were analyzed via a Goldeneye20A STR Identifiler PCR Amplification kit. Gene Mapper v3.2 software (ABI) was used to match the STR pattern with the online databases of ATCC.

### Human tissue samples

Frozen colon cancer and paired surgical margin tissue samples were collected from 15 patients. The Institutional Review Board of Peking University Cancer Hospital and Institute approved this study. Informed consent was obtained from each patient prior to inclusion in this study.

### Plasmid construction and transfection

The pEZ-M35-NRF1 expression vector and pEZ-M35 empty control vector were purchased from FulenGen Co. (Guangzhou, China). The human DNA binding domain-deleted *NRF1* mutant (Δ177–284) plasmid was kindly provided by Professor Jinrong Min at Central China Normal University [29]. Transient transfection was performed with X-tremeGENE HP DNA Transfection Reagent (Roche, Mannheim, Germany). Transfection efficiency was monitored by Western blotting.

### Chemicals

DMSO and the EZH2i tazemetostat (EPZ-6438; E-7438) were purchased from MCE (New Jersey, USA), and tazemetostat was dissolved in DMSO at a stock concentration of 10 mmol/L. EZH2i GSK343 was purchased from Selleckchem (Houston, TX, USA). GSK343 was dissolved in DMSO at stock concentrations of 100 mg/mL and 10 mmol/L according to the manufacturer’s instructions.

### RNA extraction and quantitative RT‒PCR (qRT‒PCR)

Total RNA was extracted via a Direct-Zol RNA MiniPrep Kit (Tianmo Sci & Tech Develop, Beijing, China) according to the manufacturer’s instructions. cDNA was synthesized via a First-Strand cDNA Synthesis Kit (TransGen Biotech, Beijing, China). SYBR Green PCR master mix reagents (Roche, Mannheim, Germany) were used to perform qRT‒PCR with an Applied Biosystems 7500 Real-Time PCR device (Thermo Fisher, Massachusetts, USA). The samples were analyzed in triplicate, and the expression levels of the target genes were normalized to those of the *GAPDH* gene. The 2^-ΔCt^ method was used to calculate the relative expression levels. The specific primer sets used in the qRT‒PCR assay were as follows (5’-3’): gagat ggtga tggga tttc and gaagg tgaag gtcgg agt for *GAPDH* mRNA; ttgtt ggcgg aagcg tgtaa aatc and tccct agtcc cgcgc aatgagc for *EZH2* mRNA; and atgtc cgcac agaag agcaa and ttccc gccca tgctg tttat for *NRF1* mRNA.

### Western blot

The cells were collected and lysed at approximately 80% confluence. The proteins were subjected to 10% SDS‒PAGE and then transferred onto PVDF membranes. After being blocked with 5% fat-free milk at 4 °C overnight, the membranes were incubated for 1 hr at room temperature with primary antibodies against NRF1, EZH2 (1:2500; Cell Signaling Technology, USA), H3K27Me3 (1:3000; Active Motif, USA), or GAPDH (1:15000; ProteinTech, China) at room temperature for 1 hr. After being washed with PBST (PBS with 0.1% Tween 20), the membrane was incubated with a specific horseradish peroxidase-conjugated anti-rabbit/mouse IgG antibody (SE131, Solarbio, China) at room temperature for 1 hr. The signals were visualized via an enhanced chemiluminescence kit (New Cell & Molecular Biotech, China) (Supplemental file).

### Knockdown of gene expression by siRNA

For knockdown of gene expression, small interfering double-stranded RNAs (siRNAs) targeting human *NRF1* mRNAs, including siNRF#1 (sense [5’-3’], gccac agcca cacau aguatt; antisense, uacua ugugu ggcug uggctt), siNRF1#2 (sense, gcacu acgga ccaua guuatt; antisense, uaacu auggu ccgua gugctt), and scramble control RNA (sense, uucuc cgaac guguc acgutt; antisense, acgug acacg uucgg agaatt), were synthesized by Gene Pharma (Shanghai, China). When the cells reached 70–80% confluence, they were transfected with these siRNA or scramble control mixtures (siNRF1 or NC) via PEI MAX (Polysciences, PA, USA) according to the manufacturer’s instructions. The knockdown status of target gene expression was determined by Western blotting and qRT‒PCR.

### Knockout of the *NRF1* or *EZH2* genes via CRISPR-Cas9

A single guide RNA (sgRNA; 5’-aagac agggt taggt ttgga-3’ or 5’-gactt ctgtg agctc attgc-3’; from Thermo Scientific) was used to knock out the genetic sequence in exon 3 of *NRF1* or exon 2 of the *EZH2* gene. The sgRNA was inserted into the PX458 vector (Plasmid #48138, Addgene, USA) and used to transfect HCT116 or H460 cells. A flow-sorting assay was performed for green fluorescence with a FACS Calibur flow cytometer (BD Biosciences, Franklin Lakes, USA) 48 hr post-transfection. The cells were subsequently seeded on 96-well plates to select monoclonal cells. Initial identification of *NRF1-* or *EZH2-*knockout (NRF1-KO or EZH2-KO) cell clones was carried out via genomic PCR sequencing and Western blotting. The primers used were as follows (5’-3’): sense, acctc acatt cccct tttcaca; antisense, gcctg gatta ggggg taacag for *NRF1*; sense, gagta tgttt agttc caatcgt; and antisense, ctaca gcagt catta acagtt for *EZH2*. Two monoclonal cells were pooled for each experiment.

### Confocal immunofluorescence assay

Cells grown on glass cover slips were fixed with 10% neutral formalin and permeabilized with 0.5% Triton X-100/PBS for 10 min. After being blocked with 5% BSA for 60 min, the sections were incubated with an antibody against FLAG (1:400, Protein Tech, USA) and a rabbit antibody against EZH2 (1:100) at 4 °C overnight. Normal rabbit IgG was used as the negative control. Then, the slides were washed with PBST and incubated with Alexa Fluor 647-conjugated goat anti-rabbit IgG (H + L) (1:100, Beyotime Biotech, China) and FITC-labeled goat anti-mouse IgG (1:200, Beijing Zhongshan Golden Bridge Biotech, China) at room temperature for 1 h. Nuclei were counterstained with DAPI. The cells were visualized, and images were obtained with a Zeiss confocal microscope (Oberkochen, Germany).

### Luciferase reporter assay

The promoter regions of the *EZH2* gene, including -704 to -28, named EZH2-pro1; -520 to -28, named EZH2-pro2; and –714 to –200, named EZH2-pro3, were inserted into the pGL3-Basic vector (Promega) between the *KpnI* and *Bg1II* restriction sites. HCT116 cells and HEK239T cells were transfected with a Renilla luciferase plasmid, and these *EZH2* promoter reporter vectors were cotransfected with the *NRF1* expression vector or its mutant via PEI MAX (Polysciences, USA) for 24 hr. Then, the cells were washed, lysed, and evaluated sequentially for firefly luciferase and Renilla luciferase activities according to the Dual-Luciferase Reporter Assay System (Yeasen, Shanghai, China). The results obtained were normalized to Renilla luciferase activity. The average promoter activity for 3 biological replicates was calculated. These experiments were repeated 2 times.

### Chromatin immunoprecipitation assay (ChIP)

The cells were fixed with 1% formaldehyde, lysed at 37 °C for 10 min, and sonicated to obtain sheared DNA fragments of approximately 200 – 1000 bp. The chromatin was then incubated and precipitated with the NRF1 antibody or control IgG (Millipore, USA). Protein A/G-agarose beads (Roche, Mannheim, Germany) were used to collect the DNA‒protein immunocomplexes. The precipitated DNA was subsequently purified via a DNA, RNA, and protein purification kit (Macherey-Nagel, Germany). The abundance of the NRF1 antibody-precipitated *EZH2* promoter was detected via quantitative PCR (qPCR) via the primer set (5’-3’) (sense, gccgtg tgttc agcga aaga; antisense, ccgtc caatc acagg gccc).

### GST-NRF1 purification

To construct an *NRF1* expression vector, the full-length coding region of the *NRF1* gene was amplified via PCR via the primers (5’-3’) (sense, tcccc ccggg gggaa tggag gaaca cggag tgac; antisense, ccgct cgagc ggtca ctgtt ccaat gtcac cacctcc) and inserted into the region between the *Xma1* and *XhoI* restriction sites in the pGEX-4T-1 vector. The *NRF1* vector was used to transfect BL21 chemically competent cells (TransGen Biotech, Beijing, China). The GST-NRF1 protein was extracted from bacteria and purified with glutathione Sepharose beads (GE Healthcare, Sweden).

### Electrophoresis mobility shift assay (EMSA)

The GST-NRF1 protein was used to detect NRF1-DNA binding activity via EMSA. An *EZH2* promoter fragment (5’-3’) (EZH2-bio1, ttaca gcgaa ccccg ccgcc gcccg cg*cgc gcacg cgct*g ccagt; containing *the NRF1 binding motif*), its mutant not containing the NRF1 binding motif (EZH2-mut, ttaca gcgaa ccccg ccgct ggagg tcagt ccgtt ggtct gcgcc), and two other *EZH2* promoter fragments not containing the NRF1 binding motif (EZH2-bio2, atcgc gccat tgcac tccag; and EZH2-bio3, gcgcg cgggg aaacg agcgc) were synthesized and used as EMSA probes. These probes were labeled with biotin at their 3′-end. A 10 × EZH2-bio1 probe or its mutant probe without biotin labeling was used as the competitive EMSA probe to assess the specificity of NRF1-DNA binding. A chemiluminescent EMSA kit (Beyotime, Shanghai, China) was used for EMSA analysis according to the manufacturer’s instructions.

### Cell treatment

HCT116 and LoVo cells were seeded at a density of 3 × 10^4^ cells/well in 96-well plates. After being cultured for 24 hr, the cells were treated with increasing concentrations of GSK343 (0.16, 0.8, 4, 20, and 100 µM) dissolved in culture medium containing 0.001% DMSO for 72 hr or with tazemetostat at increasing concentrations of 1.85, 5.56, 16.67, 50, and 150 µM. H460 cells were treated with increasing concentrations of GSK343 (1.67, 3.33, 10, 20, and 40 µM) dissolved in culture medium.

### Cell proliferation using IncuCyte

Cells were seeded into 96-well plates (2000 cells/well, 6 wells/group) and cultured for at least 84 hr to determine the proliferation curves. The cells were photographed every 6 hr in the long-term dynamic observation platform (IncuCyte, Essen, MI, USA). Cell confluence was analyzed using IncuCyte ZOOM software (Essen, Ann Arbor, MI, USA), and phase object confluence (%) was used to generate a cell proliferation curve.

### In vivo xenograft experiment

Five-week-old female nude BALB/c mice (18–20 g) were purchased from Beijing Huafukang Bioscience (Beijing, China) and housed under specific pathogen-free conditions. For subcutaneous tumor xenografts, 1 × 10^6^ HCT116 NRF1-WT and NRF1-KO cells were suspended in 100 μL of phosphate-buffered saline (PBS) and then inoculated subcutaneously into the left/right posterior dorsal region of each mouse (*n* = 9). The mice were euthanized when the tumors reached approximately 1 cm in diameter.

For drug treatment, HCT116 NRF1-wild-type (WT) and NRF1-KO cells (2.5 × 10^6^) were suspended in 100 μL of PBS and then inoculated subcutaneously into the posterior dorsal region of each mouse. When the tumors reached a volume of approximately 50 mm^3^ (approximately 5 mm in diameter), the mice were randomly divided into groups (NRF1-WT: *n* = 4/group; NRF1-KO: *n* = 7/group) to receive DMSO (as a solvent control) or GSK343 treatment (dissolved in a solution of 2% DMSO, 40% PEG300 and 5% Tween 80). GSK343 was administered at a dose of 10 mg/kg b.w. via intraperitoneal (ip) injection five times a week for 42 days. The mice were weighed, and the tumor diameter was measured with a caliper every 2 days without blinding. The tumor volume was calculated via an empirical formula (tumor volume = 0.5 × length × width^2^). The subcutaneous tumors were surgically excised, photographed, sectioned, and fixed in 10% formalin. This study was approved by the institutional animal ethics committee. All methods were performed in accordance with the relevant guidelines and regulations.

For the survival experiment, HCT116 NRF1-WT and NRF1-KO cells (3 × 10⁶) were suspended in 100 μL of PBS and injected subcutaneously into the right dorsum of male mice (12 mice per genotype). Then, mice in both the NRF1-WT and NRF1-KO groups were randomly divided into DMSO and GKS343 groups, which continuously received intraperitoneal injections of either vehicle DMSO or GSK343 (15 mg/kg b.w.) five times per week. Mortality in each group was monitored and recorded daily. Survival curves were generated using GraphPad Prism.

### Immunohistochemical (IHC) staining

Xenograft-derived tumor IHC staining was performed on formaldehyde-fixed paraffin-embedded (FFPE) tissue blocks. Immunohistochemistry was carried out with an anti-Ki67 (Beijing Zhongshan Golden Bridge Biotech, China) antibody. Briefly, tissue slides were dewaxed in xylene, rehydrated, subjected to antigen retrieval in 10 mM citrate buffer (pH 6.0) at 98 °C for 3 min and treated with 3% H_2_O_2_ for 10 min to block endogenous peroxidase. The slides were then blocked with 5% BSA in PBS for 30 min and incubated with primary antibody overnight at 4 °C. The PBS-washed sections were further treated with Histostain™-Plus and DAB Kits and counterstained with hematoxylin. The sections were dehydrated and stabilized with mounting medium, and images were taken with an optical microscope. Under 20× magnification, the percentage of Ki67-positive cells relative to the total number of cells (Ki67-positive index) in six fields of view for each group was calculated.

### Download of publicly available RNA-seq datasets

The mRNA levels of genes associated with *EZH2* transcription were extracted from cDNA array datasets for 961 cell lines from the Cancer Cell Line Encyclopedia (CCLE, Broad, 2019) [30] and for 9889 samples from the pancancer analysis of whole genomes [31]. These datasets were downloaded from the cBioPortal database [32–34]. The coexpression analysis results were downloaded from the GEPIA2 website (gepia2.cancer-pku.cn) [35]. The drug sensitivity data for the cancer cell lines were obtained from the Genomics of Drug Sensitivity in Cancer (GDSC2) [36] and the Cancer Therapeutics Response Portal (CTRP) v2 [37].

### Statistical analysis

Statistical analysis was carried out using SPSS 22.0 software (SPSS Inc., Chicago, IL, USA). Data are presented as the mean ± SD or as the median or the median (25-75 percentiles). Statistical analysis methods included Student’s *t* test, one-way analysis of variance (ANOVA), and two-way ANOVA. All tests were two-sided. A *P* value < 0.05 was considered to indicate statistical significance. Log-rank test was used to compare overall survival between groups. All in vitro experiments were repeated at least one time.

## Results

### NRF1 upregulates *EZH2* transcription

**Fig. 1:**
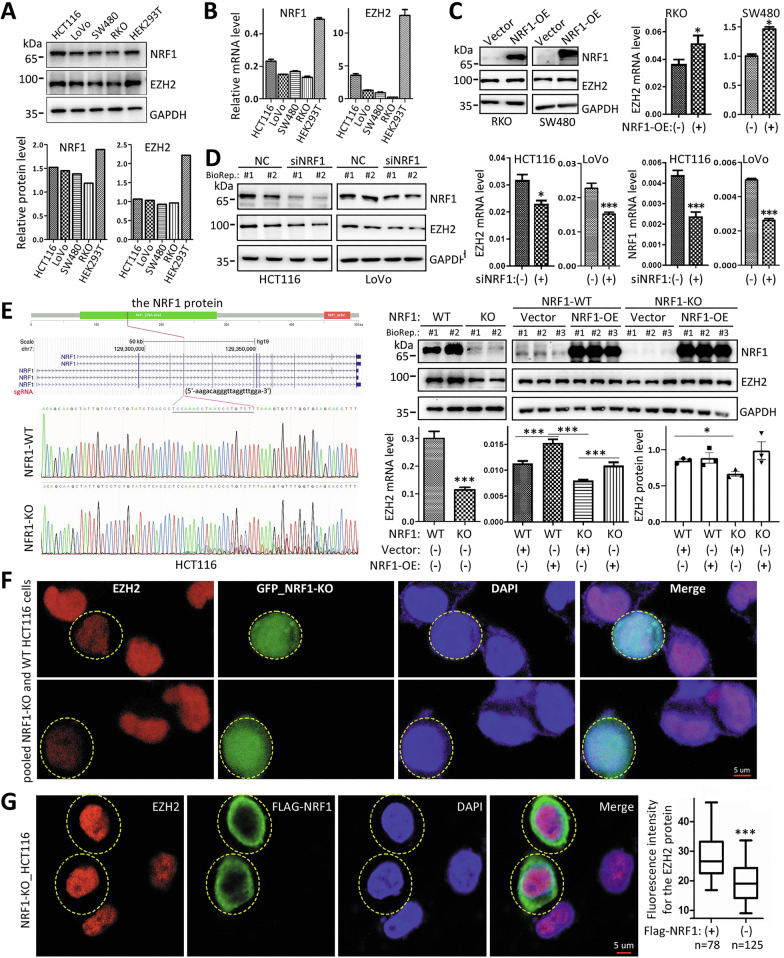
Effect of NRF1 on EZH2 expression. **A**, **B** The baseline protein and mRNA abundances of NRF1 and EZH2 in various cancer cell lines. The relative levels of these proteins are displayed in the bottom chart. **C** The mRNA and protein levels of EZH2 in RKO and SW480 cells with transient NRF1 overexpression (NRF1-OE) for 48 hr, as determined by qRT‒PCR and western blotting. The mRNA level of *EZH2* was normalized to that of *GAPDH*. **D** Effects of siRNA-mediated knockdown of *NRF1* (siNRF1) on the mRNA and protein levels of *EZH2* in HCT116 and LoVo cells for 48 hr. **E** Effect of *NRF1* knockout (NRF1-KO) via CRISPR-Cas9 and restoration of *NRF1* expression on the mRNA and protein levels of *EZH2* in HCT116 cells. The PCR-sequencing results, sgRNA-matched sites within the DNA binding domain of the NRF1 protein, and the *NRF1* exon sequence are labeled on the left. **F** Comparison of the abundance of EZH2 in pooled NRF1-WT and KO (GFP-labeled) HCT116 cells, as determined by confocal immunofluorescence microscopy analysis. **G** Comparison of the abundance of EZH2 in NRF1-KO HCT116 cells with and without the restoration of NRF1 expression by the FLAG-NRF1 vector. The data are presented as the means ± SDs for 3 biological replicates. Student’s *t* test: */***P < 0.05/0.001.

The basal levels of the NRF1 protein and mRNA in colon RKO and SW480 cancer cells were lower than those in HCT116 and LoVo cells (Fig. 1A and B). Thus, these cell lines were used in NRF1 gain- and loss-of-function experiments. The level of *EZH2* mRNA was significantly elevated in RKO and SW480 cells with transient *NRF1* overexpression (NRF1-OE) for 48 hr (Fig. 1C). Conversely, the levels of *EZH2* mRNA and protein were significantly reduced by transient siRNA-mediated knockdown of *NRF1* (siNRF1) in HCT116 and LoVo cells for 48 hr (Fig. 1D). In addition, siNRF1 decreased both the protein and mRNA levels of the *EZH2* gene in lung A549 and H1299 cancer cells but not in liver HepG2 cancer cells (Fig. S1).

When the *NRF1* exon sequence encoding the DNA binding domain was knocked out via CRISPR-Cas9 (NRF1-KO), both the mRNA and protein levels of the *EZH2* gene were markedly reduced in NRF1-KO HCT116 cells (Fig. 1E). In the rescue experiment, NRF1-OE mostly mitigated the effect of NRF1-KO on *EZH2* expression.

To confirm the effect of NRF1 on *EZH2* expression, we further performed confocal immunofluorescence microscopy analyses. We initially mixed wild-type (WT) and NRF1-KO cells, seeded them on the same slide as the NRF1 positive and negative controls, and observed an obvious decrease in EZH2 abundance in NRF1-KO cells (GFP-labeled) relative to that in NRF1-WT cells (Fig. 1F). Then, we performed a rescue experiment and found that the EZH2 level in NRF1-KO cells with FLAG-NRF1 expression was significantly greater than that in cells without FLAG-NRF1 expression (Fig. 1G). These results strongly reveal that NRF1 upregulates *EZH2* transcription in cancer cells.

### NRF1 binds the *EZH2* promoter as a transcription factor

**Fig. 2:**
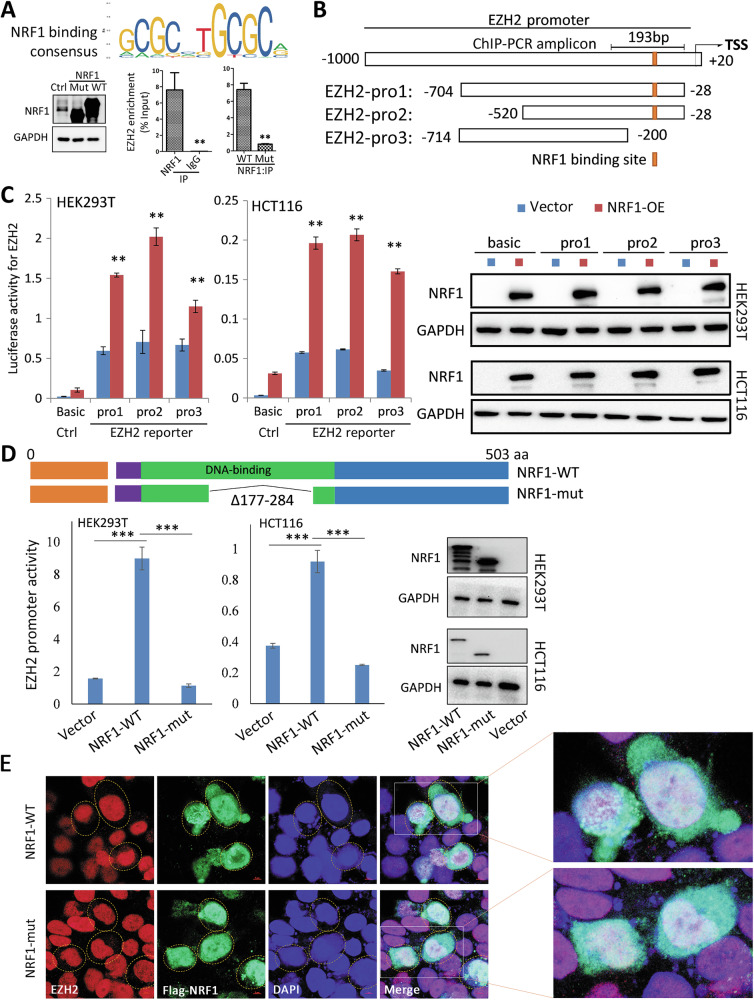
NRF1 increases EZH2 promoter activity. **A** Chromatin immunoprecipitation (ChIP)-qPCR was used to detect NRF1 binding to the *EZH2* promoter. The NRF1 binding consensus sequence predicted by Jaspar is listed at the top. **B** Schematic representation of the human EZH2 promoter, its luciferase reporter constructs, and the predicated NRF1 binding site (marked in red). **C** Comparison of the activity of various *EZH2* promoter reporters in HEK293T and HCT116 cells with and without NRF1-OE. **D** Effects of deleting the DNA binding domain (amino acids 177–284) of the NRF1 protein on *EZH2* promoter activity. **E** Subcellular location of wild-type NRF1 and the NRF1-Δ177–284 mutant in HCT116 cells. Scale bar, 5 μm. All the data are presented as the means ± SDs. Statistical analysis was performed via two-tailed Student’s *t* test. **P < 0.01; ***P < 0.001.

To determine whether NRF1 directly upregulates *EZH2* expression as a transcription factor, we searched the online promoter analysis tool Jaspar [38, 39] and identified a putative NRF1-binding site (–118 to –129 nt) in the proximal promoter flanking the transcription start site (TSS) of the *EZH2* gene (Fig. 2A, B). Chromatin immunoprecipitation (ChIP)-qPCR analysis revealed that *EZH2* promoter DNA was enriched by NRF1 in HCT116 cells (Fig. 2A). To evaluate the importance of the NRF1 binding site in regulating *EZH2* transcription by NRF1, we constructed three *EZH2* promoter reporter vectors, including the full-length *EZH2* promoter (EZH2-pro1: -28 to -704 nt) and its truncated mutants with the 184 nt deletion of the distal fragment (EZH2-pro2: -28 to -520 nt) or the 172 nt deletion of the proximal fragment (EZH2-pro3: -200 to -714 nt) not containing the NRF1 binding site (Fig. 2B). Luciferase reporter experiments revealed that the promoter activity of EZH2-pro3 was significantly lower than that of EZH2-pro1 and EZH2-pro2 in both NRF1-OE HCT116 and HEK293T cells (Fig. 2C). The baseline promoter activity of EZH2-pro3 was also lower than that of EZH2-pro1 and EZH2-pro2 in HCT116 cells. These findings reveal that the NRF1 binding site may play an important role in increasing *EZH2* transcription via NRF1.

Then, we constructed an NRF1 mutant (Δ177–284) that does not contain the DNA-binding domain. Whereas wild-type NRF1 overexpression greatly increased *EZH2* promoter activity, NRF1 mutation did not affect *EZH2* promoter activity (Fig. 2D), suggesting that the DNA-binding domain is essential for NRF1 to upregulate *EZH2*. In the confocal microscopy analysis, both the Flag-labeled WT and the Δ177–284 mutant NRF1 proteins exhibited clear and predominant nuclear localization (Fig. 2E). This finding indicates that the DBD deletion does not impair the protein’s ability to enter and accumulate in the nucleus, and the loss of promoter activation by the mutant is not due to mislocalization.

**Fig. 3:**
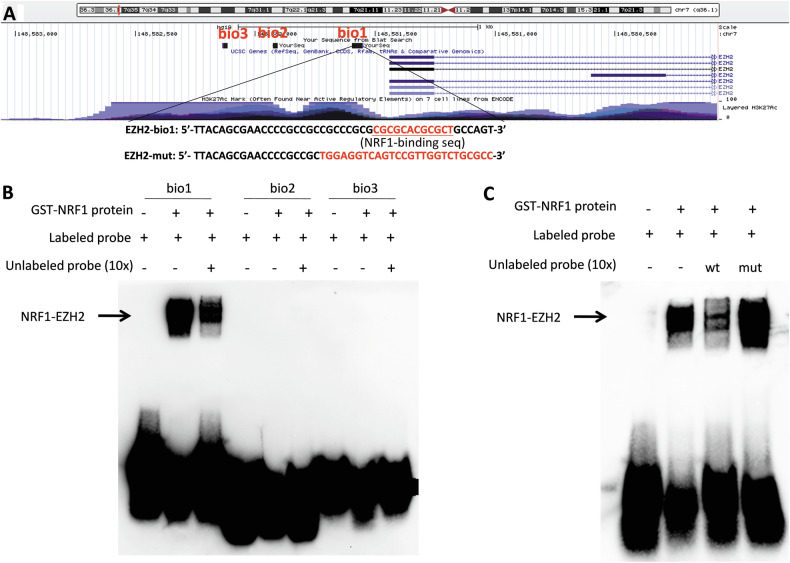
Direct binding of NRF1 to the EZH2 promoter. **A** Location and sequence information of the *EZH2* promoter probes. **B** The results of EMSA analysis to detect direct interactions between the biotin-labeled EZH2 probes containing or not containing the predicted NRF1 binding site. The EZH2 probe-GST-NRF1 binding band is indicated with arrows. **C** Comparison of differences in NRF1 binding affinity between the wild-type EZH2-bio1 probe and its mutant not containing the NRF1 binding sequence.

We further used EMSA to validate the direct binding between the NRF1 protein and the *EZH2* promoter. EMSAs revealed that the biotin-labeled *EZH2*-bio1 probe (containing the NRF1-binding site) bound the purified GST-NRF1 protein and that the interaction of the NRF1 protein with the *EZH2* promoter DNA was blocked by the addition of the “cold” *EZH2*-bio1 probe without biotin labeling. No interaction was detected between the GST-NRF1 protein and the EZH2-bio2 or EZH2-bio3 control probes (Fig. 3A, B). In addition, the “cold” EZH2-mut probe (not containing the NRF1-binding site) did not block the interaction between NRF1 and the *EZH2-*bio1 probe (Fig. 3C). These results suggest that NRF1 directly activates *EZH2* transcription as a transcription factor.

### NRF1 is a dominant cause of cancer-specific *EZH2* overexpression

There are two histone H3-lysine 27 methyltransferase genes (*EZH2* and *EZH1*) in the human genome with distinct functions. For example, *EZH1* is more abundant in nonproliferative adult organs, whereas *EZH2* expression is tightly associated with proliferation [40]. *EZH2* is frequently upregulated in many cancers (Fig. S2A), whereas *EZH1* is always downregulated in these cancers (Fig. S2B). Unexpectedly, no inverse correlation between the levels of *EZH2* and *EZH1* transcripts was observed in either cancer or normal tissues from more than ten thousand patients in the TCGA or GTEx (Fig. S2C), excluding the possibility that *EZH1* downregulation leads to *EZH2* upregulation in cancers [41].

**Fig. 4:**
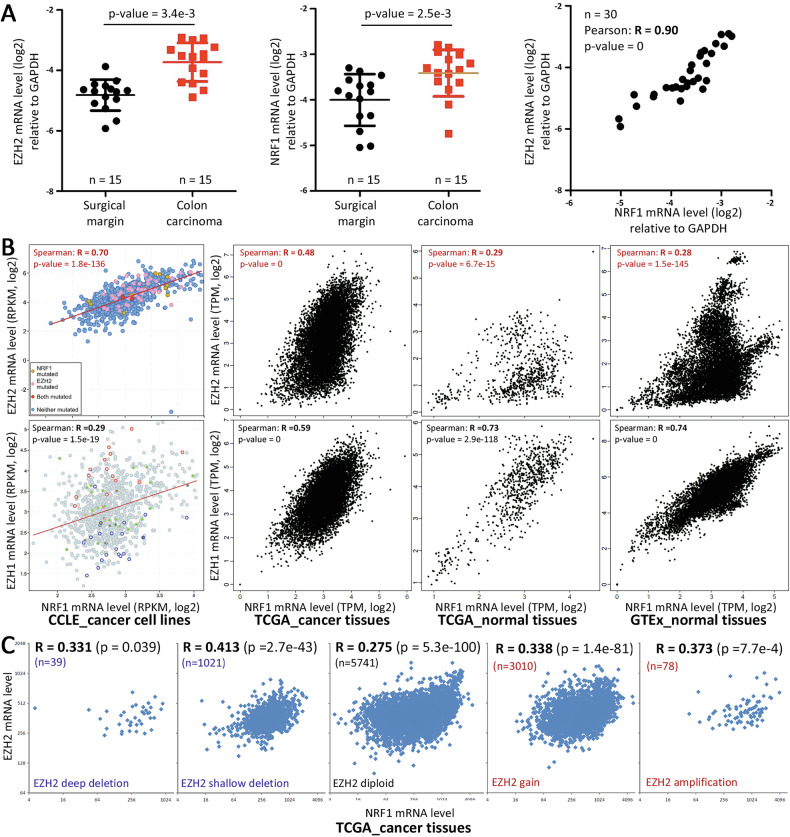
Relationship between the expression levels of *NRF1* and *EZH2*/*1* in normal and cancer tissues and cancer cell lines. **A** *EZH2* and *NRF1* mRNA levels in colon carcinoma and surgical margin tissue samples from 15 patients, as determined via qRT‒PCR. The correlation between *EZH2* and *NRF1* mRNA levels among these samples is illustrated on the left. **B** The correlation between *NRF1* and *EZH2/1* mRNA levels according to publicly available RNA-seq datasets. **C** Correlations between *EZH2* and *NRF1* mRNA levels in various cancer subgroups with different states of copy number alterations of the *EZH2* gene according to the TCGA datasets. These charts were adapted with images downloaded from the GEPIA and cBioPortal for cancer genomics websites [30, 32–35].

To evaluate the importance of NRF1 to *EZH2* overexpression in cancer cells, we compared the levels of *NRF1* and *EZH2* expression in colon carcinoma and paired surgical margin tissue samples from 15 patients. The qRT‒PCR results revealed that both of these genes were significantly upregulated in colon carcinoma tissues relative to surgical margin tissues and that *NRF1* mRNA levels were significantly correlated with *EZH2* mRNA levels (correlation coefficient (R, Pearson) = 0.90, *p* < 0.0001; Fig. 4A, right).

We further performed bioinformatics analyses. We found that the correlation coefficient (R, Spearman) between the levels of *EZH2* and *NRF1* transcripts was much greater in cancer cell lines (R = 0.70) than in cancer tissues (containing noncancer stroma cells; R = 0.48) than in normal tissues (R = 0.29 or 0.28), whereas the correlation coefficient between *EZH1* and *NRF1* transcript levels was much lower in cancer cell lines (R = 0.29) than in cancer tissues (R = 0.59) than in normal tissues (R = 0.73 or 0.74) (Fig. 4B). In addition, *EZH2* transcript levels in cancer cell lines or tissues were consistently and positively associated with the number of copies of the *NRF1* gene (Fig. S3A and S3B). EZH2 protein levels in cancer cell lines were also significantly associated with NRF1 protein levels (Fig. S3D; R = 0.47). These data suggest that NRF1 may upregulate *EZH2* in a cancer-specific fashion.

Although somatic copy number amplification caused upregulation of the *EZH2* gene (Fig. S3C), the frequency of copy number amplification of *EZH2* across cancers was very low (0.79%; 78 of 9889 samples). A positive correlation between the levels of *NRF1* and *EZH2* transcripts was consistently observed in various pancancer subgroups with and without copy number alterations in the *EZH2* gene (Fig. 4C), suggesting that NRF1 has a dominant effect on *EZH2* overexpression in cancer cells. Furthermore, *NRF1* is among the top genes whose expression is correlated mostly with *EZH2* expression and vice versa according to transcriptome datasets in the CCLE project (Fig. S4A and S4B). Some NRF1-binding proteins (including E2F1, E2F6, NFYA, and USF1) are *EZH2* promoter binding proteins. In fact, NRF1 is the only transcription factor among these top 20 *EZH2*-cotranscribing genes. These phenomena suggest that NRF1 may be a master transcription factor for the *EZH2* gene.

To investigate the role of NRF1 in colon cancer, we detected the effect of NRF1-KO on the proliferation of HCT116 cells in vitro and in vivo. The results of long-term dynamic monitoring of live cells revealed that compared with NRF1-WT, NRF1-KO significantly inhibited cell proliferation (Fig. S5A). Furthermore, we established a tumor xenograft mouse model by subcutaneously implanting NRF1-WT and NRF1-KO HCT116 cells into nude mice. The results revealed that NRF1-KO cell-derived xenografts were observed in only one of these mice (1/9), whereas NRF1-WT cell-derived xenografts were observed in all nine mice (9/9) (Fig. S5B, *P* < 0.0001). In addition, the stable restoration of *EZH2* expression significantly mitigated the proliferation of NRF1-KO HCT116 cells (Fig. S5C) and the growth of tumors derived from these cells (Fig. S5D). Similarly, stable NRF1-OE promoted the proliferation of RKO cells (Fig. S6A, S6B).

Furthermore, we found that NRF1-OE not only increased EZH2 expression but also increased the global level of H3K27Me3 in both normal colon fibroblast CCD-18Co and colon cancer RKO cell lines (Fig. S6A and Fig. S10), suggesting that NRF1 increases EZH2 activity. Collectively, these results indicate that NRF1 indeed promotes tumor growth.

### NRF1 increases the sensitivity of cancer cells to EZH2 inhibitors

GSK343 is an EZH2i. By analyzing the GDSC2 datasets [36] and Cancer Cell Line Encyclopedia (CCLE) datasets [30], we found that among the transcription factor ChIP-seq clusters from ENCODE with factorbook motifs (Fig. S4C) [39], information on both the area under curve (AUC) of GSK343 and the mRNA levels was available for 71 transcription factors that can bind the *EZH2* promoter in human cancer cell lines (*n* = 845) and that the correlation coefficient between the levels of the *NRF1* transcript and the AUC of GSK343 was among the top three (Fig. S7A). A similar relationship was also observed between the sensitivity of cancer cell lines (*n* = 750) to BRD-K62801835, another EZH2i, by analyzing the CTRPv2 datasets (Fig. S7B) [37].

In addition, the levels of both *EZH2* and *NRF1* transcripts were inversely correlated with the half maximal inhibitory concentration (IC50) of GSK343 (R = -0.267 and -0.320, respectively) (Fig. S8A). When these cell lines were subclassified as *NRF1* or *EZH2* expression-high or -low according to the median mRNA level, the GSK343-IC50 of the *NRF1* or *EZH2* expression-high cancer cell lines was significantly lower than that of the *NRF1* or *EZH2* expression-low cancer cell lines (Fig. S8B). Notably, the GSK343-IC50 of cell lines with high *NRF1* expression was significantly lower than that of cells with low *NRF1* expression among the cell lines with high *EZH2* expression, whereas no significant difference in the GSK343-IC50 was detected between *NRF1*-high cell lines and *NRF1*-low cell lines among the cell lines with low *EZH2* expression (Fig. S8C). These results suggest that NRF1 may affect the sensitivity of cancer cells to GSK343 through the upregulation of *EZH2*.

**Fig. 5:**
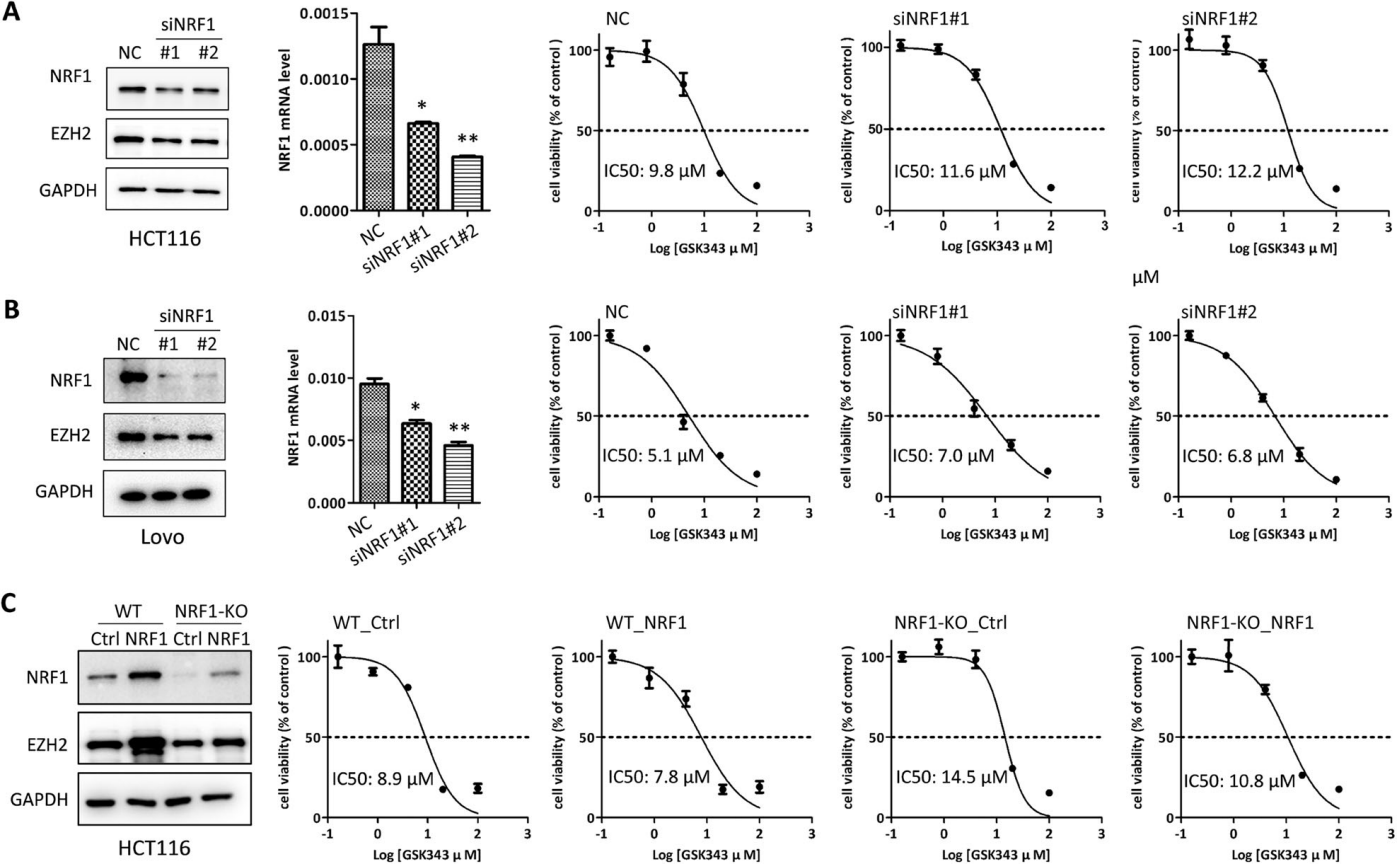
Effect of changes in *NRF1* expression on the dose‒response curve and viability of cells treated with GSK343. **A**, **B** Effect of siNRF1 on the sensitivity of HCT116 and LoVo cells to GSK343. The status of *NRF1* knockdown was monitored by Western blotting and qRT‒PCR. **C** Effect of NRF1-KO and restoration of *NRF1* expression on the sensitivity of HCT116 cells to GSK343. The status of *NRF1* restoration was monitored by Western blotting. The GSK343-IC50 values are labeled. Student’s *t* test, *P < 0.05; **P < 0.01.

We further found that the GSK343-IC50 was increased by siNRF1 in both HCT116 cells (9.8–11.6 or 12.2 μM) and LoVo cells (5.1–7.0 or 6.8) (Fig. 5A, B). We also found that the GSK343-IC50 was increased by NRF1-KO in HCT116 cells (8.9 to 14.5). NRF1-OE clearly mitigated the effect of NRF1-KO (14.5 to 10.8) on the GSK343-IC50 in the rescue experiment (Fig. 5C). Similar effects of changes in *NRF1* expression on the sensitivity of another EZH2i, tazemetostat (EPZ-6348), were also detected (Fig. S9). Furthermore, NRF1-OE decreased the GSK343-IC50 in RKO cells (Fig. S6C).

**Fig. 6:**
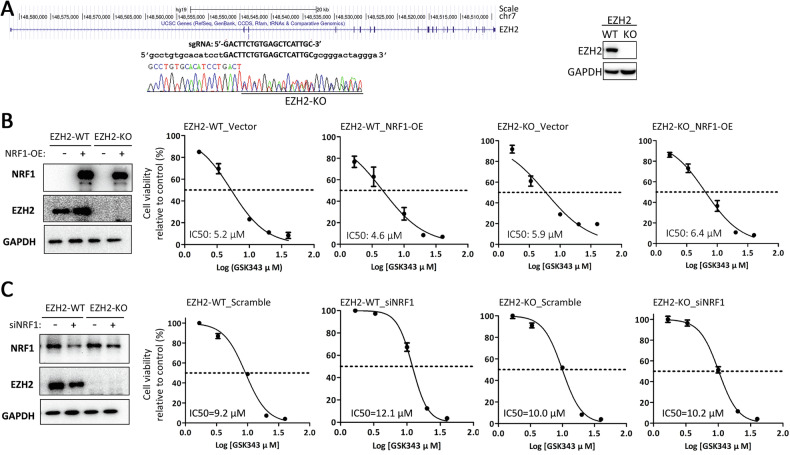
Effect of *EZH2* knockout and *NRF1* expression changes on the dose‒response curve and viability of H460 cells in response to GSK343. **A** Location of the sgRNA and characterization of *EZH2* knockout, as determined by PCR sequencing (left) and Western blotting (right). **B**, **C** Effects of NRF1-OE and siNRF1 on the sensitivity of EZH2-WT and EZH2-KO cells to GSK343. *NRF1* expression and knockdown were monitored by Western blotting. GSK343-IC50 values are labeled.

To evaluate whether NRF1 affects the sensitivity of cancer cells to EZH2is through the upregulation of *EZH2*, we knocked out the *EZH2* gene (EZH2-KO) in H460 cells (Fig. 6A) and used these cells to determine whether EZH2-KO cancels the effects of NRF1 on the sensitivity of cancer cells to EZH2is. EZH2-KO alone weakly increased the GSK343-IC50 (5.2 to 5.9 or 9.2 to 10.0). While NRF1-OE or siNRF1 decreased or increased the GSK343-IC50 only in EZH2-WT cells, such effects were not detected in EZH2-KO cells (Fig. 6B, C), demonstrating that the effects of NRF1 expression changes on the GSK343-IC50 were dependent on the effect of NRF1 on *EZH2* transcription.

**Fig. 7:**
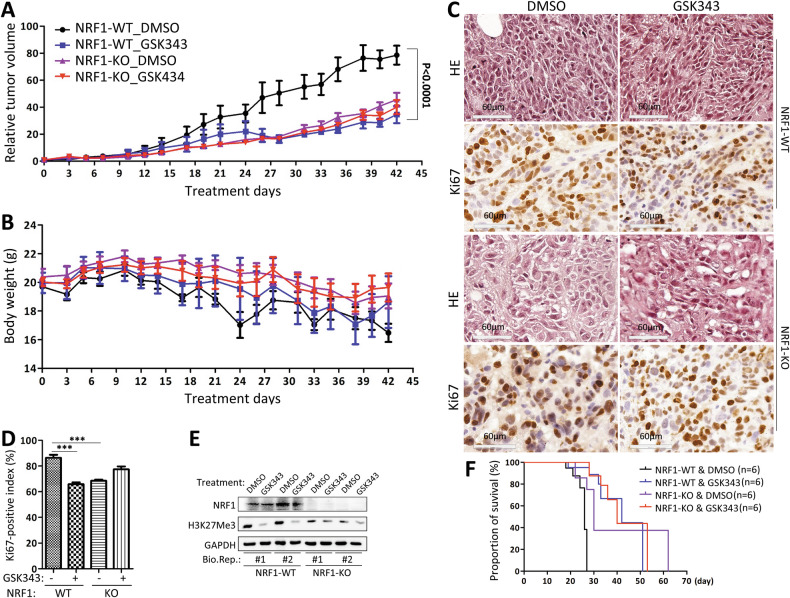
Effect of NRF1-KO and the EZH2i GSK343 on the proliferation of HCT116 cells in vivo. GSK343 and the DMSO control were injected into nude mice (WT-DMSO: *n* = 4; WT-GSK343: *n* = 4; NRF1-KO_DMSO: *n* = 7; NRF1-KO_GSK343: *n* = 7). **A** Relative tumor volumes. **B** Mouse body weight. **C**, **D** Histological HE and Ki67-stained tissue images of representative tumor xenografts. The Ki67-positive index is presented as the mean ± SD. Student’s *t* test, ***P < 0.001. **E** Western blotting images for the detection of H3K27Me3 abundance in NRF1-WT or NRF1-KO cell-derived xenografts from mice with and without GSK343 treatment. **F** Overall survival curves of mice bearing NRF1-WT or NRF1-KO xenografts in the DMSO control and GSK343 groups (GSK343: 15 mg/kg b.w., five times per week, continuously). Log-rank analysis, *P* < 0.001 or 0.212 between the DMSO and GKS343 groups for mice bearing NRF1-WT or NRF1-KO xenografts.

To confirm the impact of NRF1 on the sensitivity of cancer cells to EZH2i in vivo, we established xenograft mouse models by subcutaneously implanting NRF1-WT and NRF1-KO HCT116 cells into nude mice. When the tumors became palpable (approximately 50 mm^3^), the mice were treated with DMSO (as a solvent control) or GSK343 at a dose of 10 mg/kg. After 42 days of GSK343 treatment, the tumors in the control group reached the predetermined endpoint. Strikingly, GSK343 treatment was much more effective than DMSO treatment for NRF1-WT tumors, whereas there were no significant differences in the growth of NRF1-KO tumors between the GSK343 group and the DMSO group (Fig. 7A), while all the mice exhibited a decrease in body weight, particularly those in the NRF1-WT group (Fig. 7B). The growth of NRF1-WT tumors was greater than that of NRF1-KO tumors in the mice treated with DMSO. This difference was not observed in the mice treated with GSK343. Moreover, the Ki67 staining results were consistent with the tumor volumes (Fig. 7C, D). The baseline H3K27Me3 level in NRF1-WT cell-derived xenografts was greater than that in NRF1-KO cell-derived xenografts in DMSO-treated mice. The H3K27Me3 level in NRF1-WT xenografts was markedly decreased by GSK343 treatment, whereas it was only weakly decreased in NRF1-KO xenografts (Fig. 7E).

The results of the mouse survival experiment further indicated that NRF1-WT xenografts were more sensitive to GSK343 than NRF1-KO xenografts. The overall survival of mice bearing NRF1-WT tumors was significantly prolonged by GSK343 treatment (*P* < 0.001), whereas that of mice bearing NRF-KO tumors was not (*P* = 0.212; Fig. 7F).

Taken together, these in vivo results confirmed that NRF1 increased the sensitivity of cancer cells to EZH2i. *NRF1* loss abolished the effect of EZH2i treatment on the growth of tumors.

## Discussions

EZH2 is a cancer driver and therapeutic target that is consistently upregulated in many cancers. How EZH2 is upregulated in cancer cells and why most cancer patients cannot benefit from EZH2i treatment are not clear [21, 22]. NRF1 is a typical transcription factor [25, 42–44]. In this study, via bioinformatics analyses and systemic experiments, we found that NRF1 is a master transcription factor of the *EZH2* gene and that *NRF1* upregulation is a determinant of *EZH2* overexpression in human cancers. Most importantly, our findings demonstrate that active *NRF1* expression significantly increases the sensitivity of colon cancer cells to EZH2i in an EZH2-dependent manner and that NRF1 loss disrupts the anticancer effect of EZH2i treatment.

EZH2 is the key catalytic subunit of PRC2, and its overexpression drives cancer development via the H3K27me3 and nonhistone protein methylation pathways [1, 10]. There are numerous reports on target genes of the EZH2 protein and its posttranslational modifications, including phosphorylation, acetylation, ubiquitination, GlcNAcylation, and SUMOylation [45]. Somatic copy number amplification of the *EZH2* gene may partially account for *EZH2* overexpression in a small proportion of human cancers because the frequency of *EZH2* amplification is less than 1% in cancer samples in the TCGA project. We found that *NRF1* knockdown markedly decreases EZH2 abundance in many cancer cell lines. However, *NRF1* overexpression did not increase EZH2 abundance. The extensive upregulation of EZH2 and NRF1 across cancer cells may account for this conflict. The EZH2 abundance in cancer cells is too high to increase. The fact that *NRF1* overexpression increased EZH2 abundance in NRF1-KO cells supports that NRF1 is a master transcription factor for the *EZH2* gene.

EZH2 is reportedly regulated by several transcription factors. For example, the transcription factors BRD4 and E2Fs induce *EZH2* transcriptional activation in bladder tumors [46, 47]. C-MYC promotes *EZH2* overexpression in leukemia and prostate cancers [48, 49]. STAT3 upregulates *EZH2* transcriptional activation and is associated with poor prognosis in patients with gastric cancer [50]. However, the determinants and exact mechanisms underlying *EZH2* overexpression in human cancers are not clear. Both the *EZH2* and *NRF1* genes are overexpressed in a variety of cancers [7, 12, 51–53], and we speculate that NRF1 may play an important role in this process. For this purpose, we analyzed RNA-seq datasets from the CCLE and TCGA databases and found that the transcription levels of *NRF1* and *EZH2* were mostly correlated with each other in cancers. Notably, the correlation between *NRF1* and *EZH2* transcription gradually decreased from cancer cell lines to cancer tissues, noncancerous tissues from cancer patients, and normal human tissues, suggesting a tumor-specific effect of NRF1 on *EZH2* overexpression. In contrast, the transcriptional correlation between *NRF1* and *EZH1* (normally expressed in nonproliferative adult cells) gradually increased from cancer cells to normal cells. Our bioinformatics and experimental findings indicate that NRF1 may be a master regulator of *EZH2* and that its overexpression may be the determinant of *EZH2* overexpression in human cancers.

While no direct binding signal was detected between NRF1 and the EZH2-pro3 probe without the NRF1 binding sequence in the EMSA analysis, we found that NRF1-OE still weakly increased the promoter activity of the EZH2-pro3 reporter, suggesting that there may be other pathways by which NRF1 affects EZH2 expression. Several *EZH2* transcription factors, such as E2F1 and E2F6, are encoded by NRF1-binding genes, according to ENCODE datasets [25]. Whether these transcription factors contribute to *EZH2* regulation by NRF1 is worth further study.

As an epigenetic silencer, EZH2 plays essential roles in multiple biological processes. *EZH2* overexpression is common in human cancers and is associated with aggressiveness, poor prognosis, and recurrence as a cancer driver [6, 54]. While EZH2 is regularly recognized as an oncogene, it may also function as a tumor suppressor in some cancers in a cellular context-dependent manner, in which inactivating loss-of-function mutations of *EZH2* have been identified, underscoring caveats in applying therapeutic EZH2 inhibitors [6, 55–59].

Researchers have synthesized various small EZH2i chemicals, some of which have entered clinical trials or been approved for clinical treatment [60–62]. For example, CPI-1205 [63], GSK343 [64], GSK126 [18], and SHR2554 [65] are in clinical trials. Tazemetostat and valemetostat have been marketed for the treatment of rare adult sarcomas in the USA [14] and T-cell leukemia/lymphoma in Japan [16]. Unfortunately, only a limited proportion of cancer patients benefit from treatment with EZH2i. Thus, predictors of the sensitivity of cancer patients to treatment with EZH2i are urgently needed. In this study, we analyzed the correlation between the levels of *NRF1* and/or *EZH2* transcripts and the sensitivity of cancer cell lines to GSK343 in GDSC2 datasets. We found that the expression levels of both the *NRF1* and *EZH2* genes were inversely correlated with the IC50 of GSK343, and the cells with high *NRF1* and *EZH2* coexpression were more sensitive to GSK343 than were the cells with high individual *NRF1* or *EZH2* expression or low *NRF1* and *EZH2* coexpression. In addition, the correlation coefficient of *NRF1* mRNA with the AUC of GSK343 is the third highest among 71 transcription factor candidates for *EZH2*, according to the public ENCODE and GDSC2 data [36, 39]. Knockdown or knockout of *NRF1* decreased the sensitivity of cancer cells to GSK343 and tazemetostat in an EZH2-dependent manner. Similar phenomena were also observed for another EZH2i, BRD-K62801835, according to the CTRCv2 data [37]. Our in vivo experiment also revealed that NRF1-KO greatly decreased the total H3K27Me3 level in HCT116 xenografts and the sensitivity of xenografts to GSK343 treatment. These results suggest that *NRF1* overexpression increases the sensitivity of *EZH2-*expressing cells to EZH2is and may be a potential biomarker for predicting the therapeutic efficacy of EZH2is. Further clinical trials are warranted to study the feasibility of using high *NRF1* and *EZH2* coexpression as a combined biomarker for predicting the therapeutic effects of EZH2is. The causes of *NRF1* overexpression in cancer cells and whether NRF1 itself is a therapeutic target are also worthy of study.

In conclusion, we found that NRF1, as a transcription factor, predominantly upregulates the transcription of the *EZH2* gene. *NRF1* overexpression not only causes *EZH2* overexpression in cancer cells but also increases the sensitivity of cancer cells to EZH2i. High *NRF1* and *EZH2* coexpression is a potential combined biomarker for predicting the therapeutic effects of EZH2is.

## Supplementary information

Supporting information

Raw images for Western blotting

## Data Availability

All data are included in the paper and supporting files.
