# Supplementary material for: NRF1 predominantly causes EZH2 overexpression in cancer cells
Source: Cell Death Dis. 2026 May 16;17(1):625. doi: 10.1038/s41419-026-08861-4 (PMC13346543; doi:10.1038/s41419-026-08861-4)
Supplement: Supplementary file 1 — Supporting information [file 41419_2026_8861_MOESM1_ESM.pdf]

## Supporting information

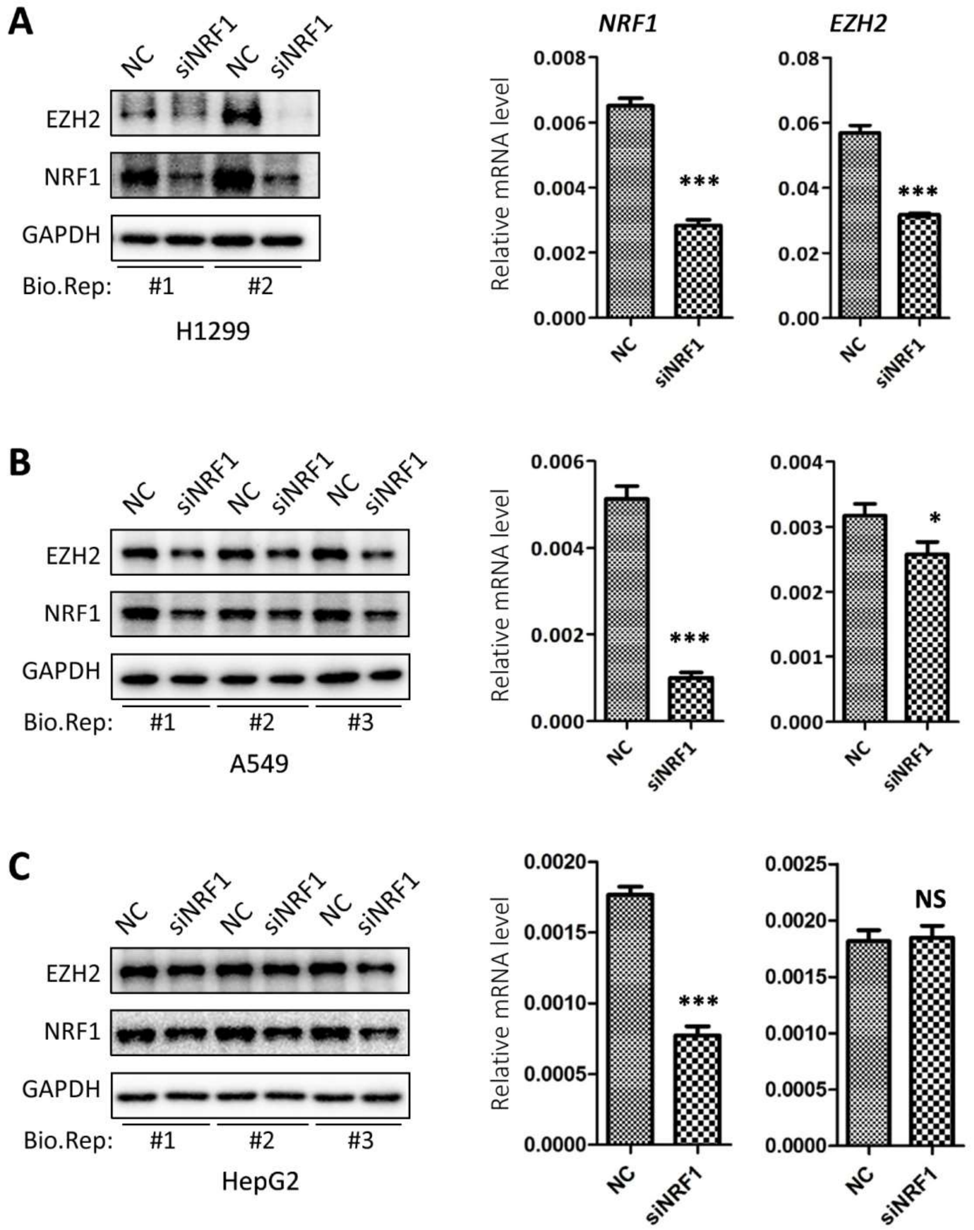


**Figure S1. Effect of *NRF1* knockdown on *EZH2* expression.** The effects of siRNA-mediated knockdown of *NRF1* (siNRF1) on the protein and mRNA levels of *EZH2* in H1299 cells (**A**), A549 cells (**B**) and HepG2 cells (**C**) 48 hrs post-transfection. The protein levels were detected by Western blotting (left images). The mRNA levels were detected via qRT–PCR. All the data are presented as the means ± SDs. Statistical analysis was performed via two-tailed Student's t test. *P < 0.05; ***P < 0.001

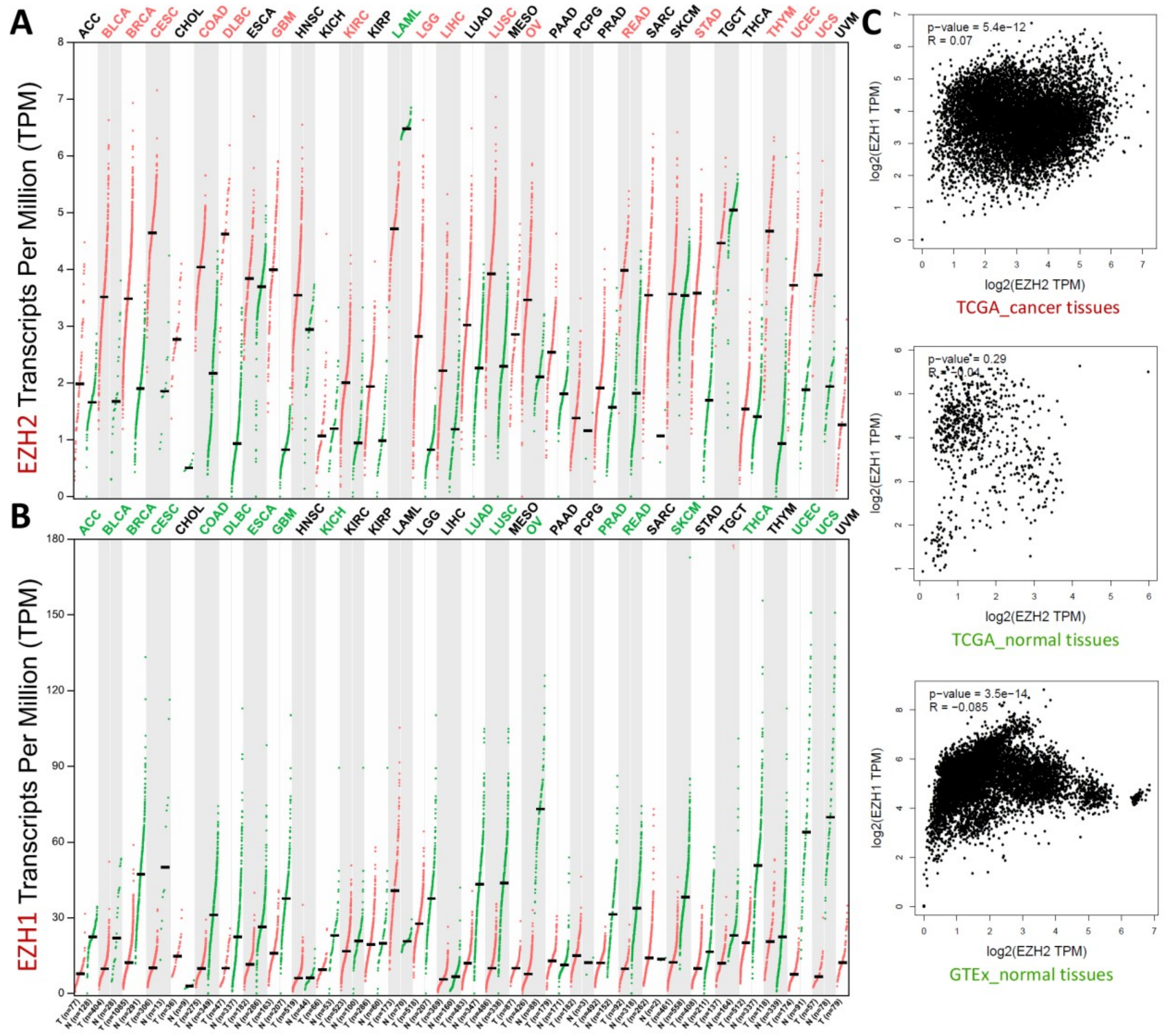


**Figure S2. Comparisons of the expression states of *EZH2* and *EZH1* genes in human cancer and normal tissues**. The levels of *EZH2* (**A**) and *EZH1* (**B**) transcripts in different types of cancer tissues and paired normal tissues in the TCGA project (35). The red or green dots represent the mRNA levels in cancer or normal tissue samples. The red and green abbreviations represent cancer types in which the EZH2 or EZH1 genes are significantly upregulated or downregulated, respectively. (**C**) Correlations between EZH2 mRNA levels and EZH1 mRNA levels in human cancer and normal tissues. These charts were adapted with images downloaded from the GEPIA website.

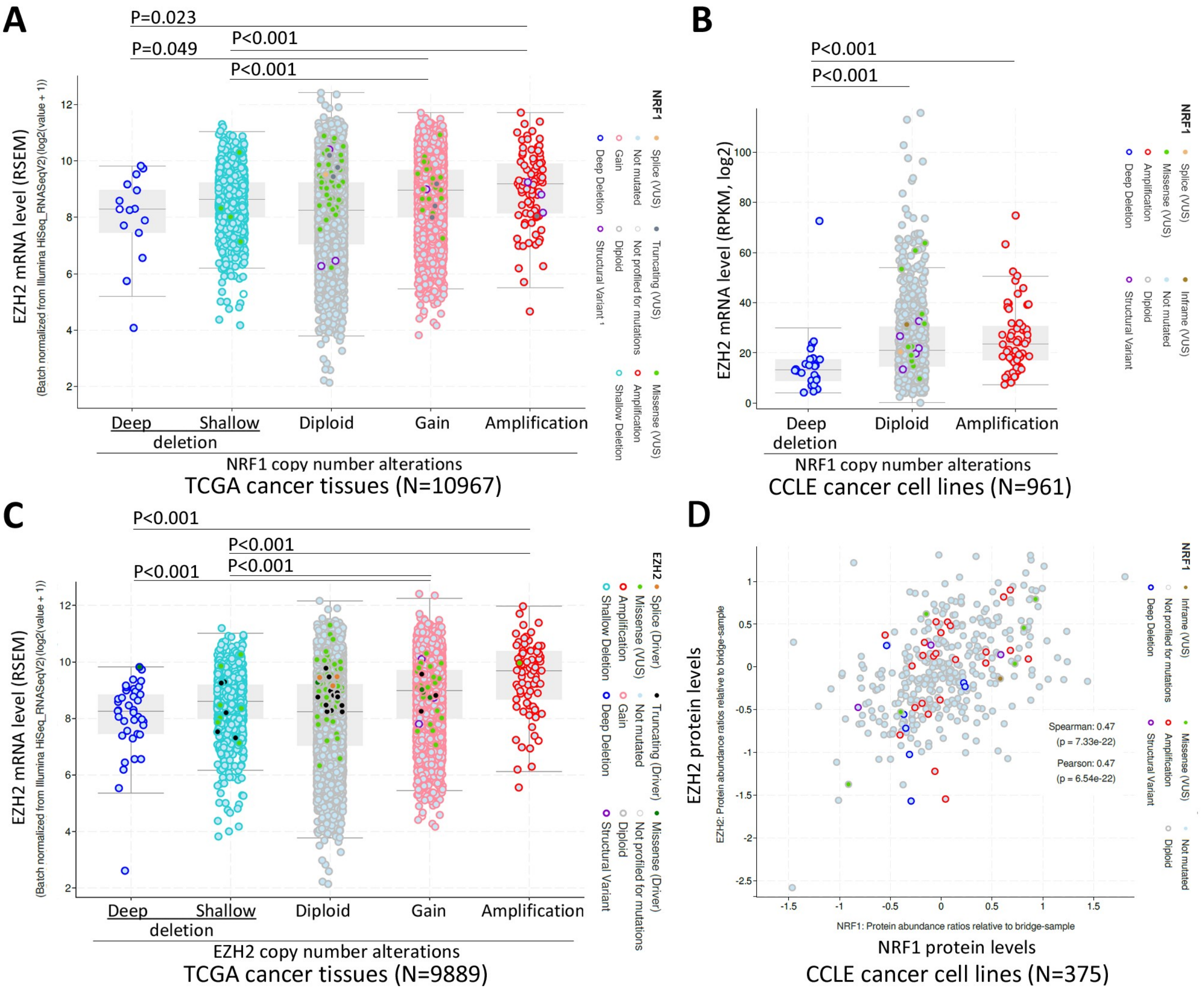


**Figure S3. Comparison of the levels of *EZH2* expression in cancer tissues with different copy number alterations and the expression levels of the *NRF1* and *EZH2* genes according to the TCGA and CCLE datasets.** (**A** and **B**) Correlations between *EZH2* mRNA levels and *NRF1* copy number in cancer tissues and cancer cell lines. (**C**) Correlations between the mRNA levels and copy numbers of the *EZH2* gene in cancer tissues. (**D**) Correlations between the levels of the EZH2 and NRF1 proteins in cancer cells in CCLE (Broad 2019), as determined by mass spectrometry. This chart was adapted with images downloaded from the cBioPortal for cancer genomics website (32-34). The P value between two target groups in Mann-Whitney test is labeled.

**A** [top 20 genes mostly coexpressing with NRF1]

| Gene | Cytoband | Biological function | Spearman's Correlation | p-Value | q-Value ▲ |
|---|---|---|---|---|---|
| **HCFC1** | Xq28 | transcriptional coregulator | 0.701 | 6.96e-137 | **2.19e-132** |
| **EZH2** | 7q36.1 | histone methyltransferase | 0.700 | 1.79e-136 | **2.82e-132** |
| **SAFB** | 19p13.3 | DNA-binding protein | 0.680 | 5.73e-126 | **6.01e-122** |
| **CTCF** | 16q22.1 | chromatin assembly | 0.661 | 1.22e-116 | **9.60e-113** |
| **ILF3** | 19p13.2 | biogenesis of circular RNAs | 0.660 | 3.03e-116 | **1.91e-112** |
| **CHAF1A** | 19p13.3 | nucleosome assembly | 0.659 | 5.74e-116 | **3.01e-112** |
| **ELAVL1** | 19p13.2 | RNA-binding | 0.659 | 7.29e-116 | **3.28e-112** |
| **SF1** | 11q13.1 | pre-mRNA splicing | 0.635 | 3.36e-105 | **1.32e-101** |
| **RBM10** | Xp11.3 | RNA binding and splicing | 0.631 | 1.59e-103 | **5.56e-100** |
| **UBTF** | 17q21.31 | transcription factor | 0.631 | 2.91e-103 | **9.17e-100** |
| **PAXIP1** | 7q36.2 | DNA damage response | 0.623 | 3.50e-100 | **1.00e-96** |
| **DNMT1** | 19p13.2 | DNA methylation | 0.620 | 4.76e-99 | **1.25e-95** |
| **E2F2** | 1p36.12 | transcription factor | 0.617 | 1.23e-97 | **2.97e-94** |
| **RBMX** | Xq26.3 | post-transcriptional processes | 0.615 | 5.36e-97 | **1.20e-93** |
| **RBM14** | 11q13.2 | nuclear coactivator | 0.613 | 2.65e-96 | **5.56e-93** |
| **CASP2** | 7q34 | cellular apoptosis | 0.613 | 3.41e-96 | **6.72e-93** |
| **ESPL1** | 12q13.13 | chromosome segregation | 0.611 | 2.28e-95 | **4.23e-92** |
| **MCM7** | 7q22.1 | DNA replication | 0.610 | 4.87e-95 | **8.52e-92** |
| **DOT1L** | 19p13.3 | histone methyltransferase | 0.610 | 5.64e-95 | **9.34e-92** |
| **SRRT** | 7q22.1 | RNA binding | 0.609 | 1.25e-94 | **1.96e-91** |
| **CPSF6** | 12q15 | 3' RNA cleavage | 0.609 | 1.54e-94 | **2.31e-91** |
| **HNRNPL** | 19q13.2 | Splicing factor binding | 0.608 | 2.55e-94 | **3.65e-91** |
| **TMPO** | 12q23.1 | organization of the nucleus | 0.608 | 3.53e-94 | **4.83e-91** |
| **FBXO5** | 6q25.2 | protein ubiquitination | 0.605 | 4.93e-93 | **6.46e-90** |
| **TIMELESS** | 12q13.3 | DNA replication; circadian clock | 0.604 | 8.43e-93 | **1.06e-89** |

**B** [top 20 genes mostly coexpressing with EZH2]

| Gene | Cytoband | Biological function | Spearman's Correlation | p-Value | q-Value ▲ |
|---|---|---|---|---|---|
| **PAXIP1** | 7q36.2 | DNA damage response | 0.733 | 3.27e-156 | **1.03e-151** |
| **CASP2** | 7q34 | cellular apoptosis | 0.705 | 4.28e-139 | **6.73e-135** |
| **XRCC2** | 7q36.1 | DNA repair | 0.700 | 1.30e-136 | **1.37e-132** |
| **NRF1** | 7q32.2 | transcription factor | 0.700 | 1.79e-136 | **1.41e-132** |
| **SRSF10** | 1p36.11 | RNA splicing | 0.668 | 5.27e-120 | **3.32e-116** |
| **NUP205** | 7q33 | nuclear pore complex | 0.657 | 7.45e-115 | **3.91e-111** |
| **MCM7** | 7q22.1 | DNA replication | 0.657 | 1.07e-114 | **4.80e-111** |
| **HMGB2** | 4q34.1 | DNA end-joining processes | 0.638 | 2.24e-106 | **8.80e-103** |
| **NCAPG2** | 7q36.3 | chromosome assembly | 0.628 | 5.47e-102 | **1.75e-98** |
| **TMPO** | 12q23.1 | organization of the nucleus | 0.628 | 5.55e-102 | **1.75e-98** |
| **ZNF212** | 7q36.1 | transcription coregulator | 0.623 | 4.41e-100 | **1.26e-96** |
| **E2F2** | 1p36.12 | transcription factor | 0.619 | 1.15e-98 | **3.02e-95** |
| **RBMX** | Xq26.3 | post-transcriptional processes | 0.619 | 1.71e-98 | **4.15e-95** |
| **DBF4** | 7q21.12 | DNA replication | 0.618 | 6.28e-98 | **1.41e-94** |
| **MCM6** | 2q21.3 | DNA replication | 0.614 | 2.43e-96 | **5.11e-93** |
| **TIMELESS** | 12q13.3 | DNA replication; circadian clock | 0.611 | 3.32e-95 | **6.53e-92** |
| **CHAF1A** | 19p13.3 | nucleosome assembly | 0.604 | 1.12e-92 | **2.08e-89** |
| **MCM3** | 6p12.2 | DNA replication | 0.604 | 1.65e-92 | **2.89e-89** |
| **HCFC1** | Xq28 | transcriptional coregulator | 0.603 | 4.27e-92 | **7.08e-89** |
| **FBXO5** | 6q25.2 | protein ubiquitination | 0.602 | 4.87e-92 | **7.67e-89** |
| **CPSF6** | 12q15 | 3' RNA cleavage | 0.602 | 5.20e-92 | **7.79e-89** |
| **RSBN1L** | 7q11.23 | lysine-specific demethylase | 0.602 | 6.18e-92 | **8.85e-89** |
| **ILF3** | 19p13.2 | biogenesis of circular RNAs | 0.598 | 1.48e-90 | **2.03e-87** |
| **SAFB** | 19p13.3 | DNA-binding protein | 0.593 | 1.68e-88 | **2.20e-85** |
| **DNMT1** | 19p13.2 | DNA methylation | 0.593 | 1.95e-88 | **2.45e-85** |

**C**

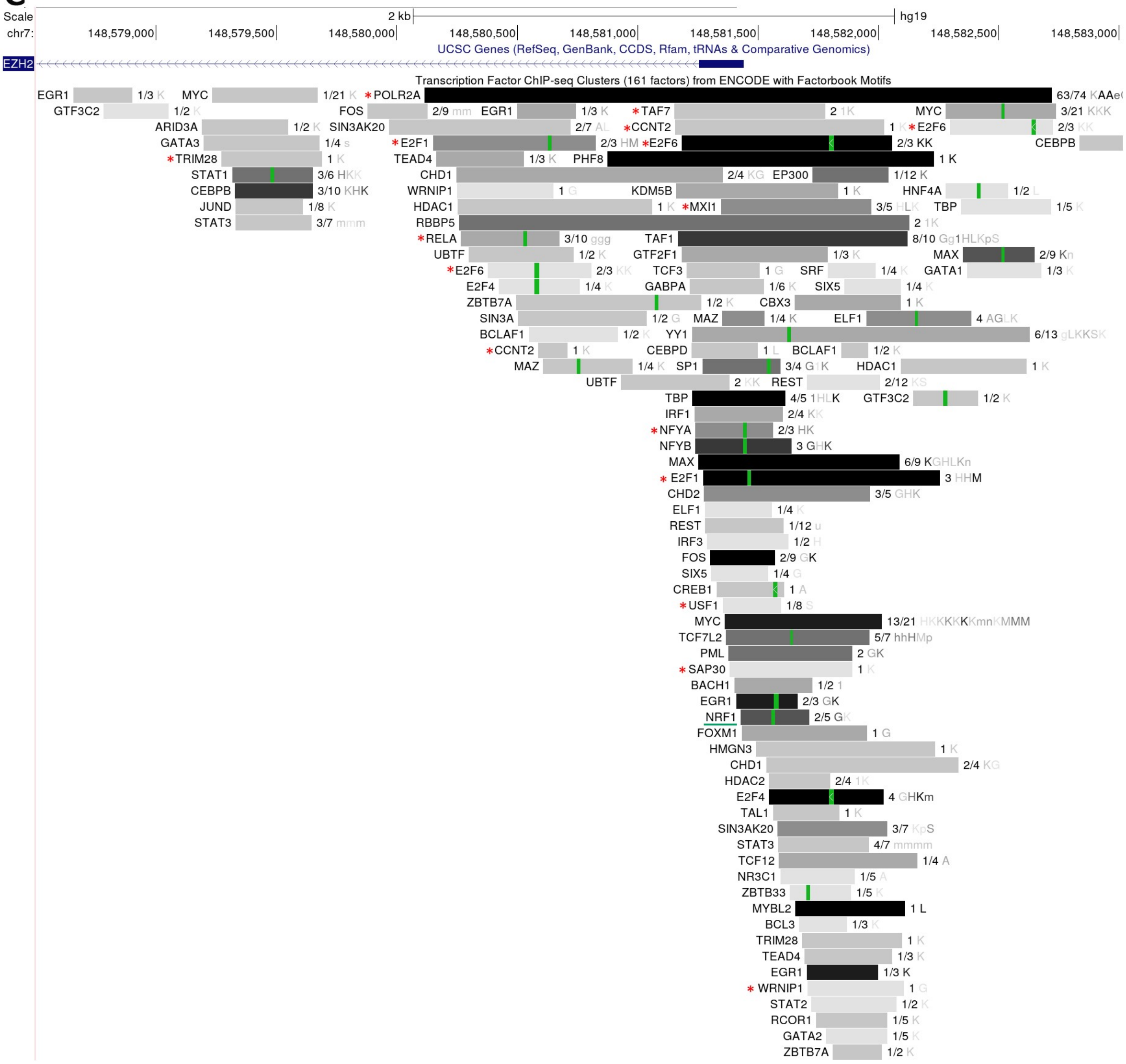

**Figure S4. Top 20 genes cotranscribed with the *NRF1* and *EZH2* genes in 921 cancer cell lines in the Cancer Cell Line Encyclopedia (CCLE)** (35) **(**A **and** B**) and graphic views of the transcription factor (TF) ChIP-seq cluster from ENCODE with factorbook motifs around the *EZH2* promoter** (C**);** *: EZH2 promoter binding proteins encoded by NRF1 binding genes (25).

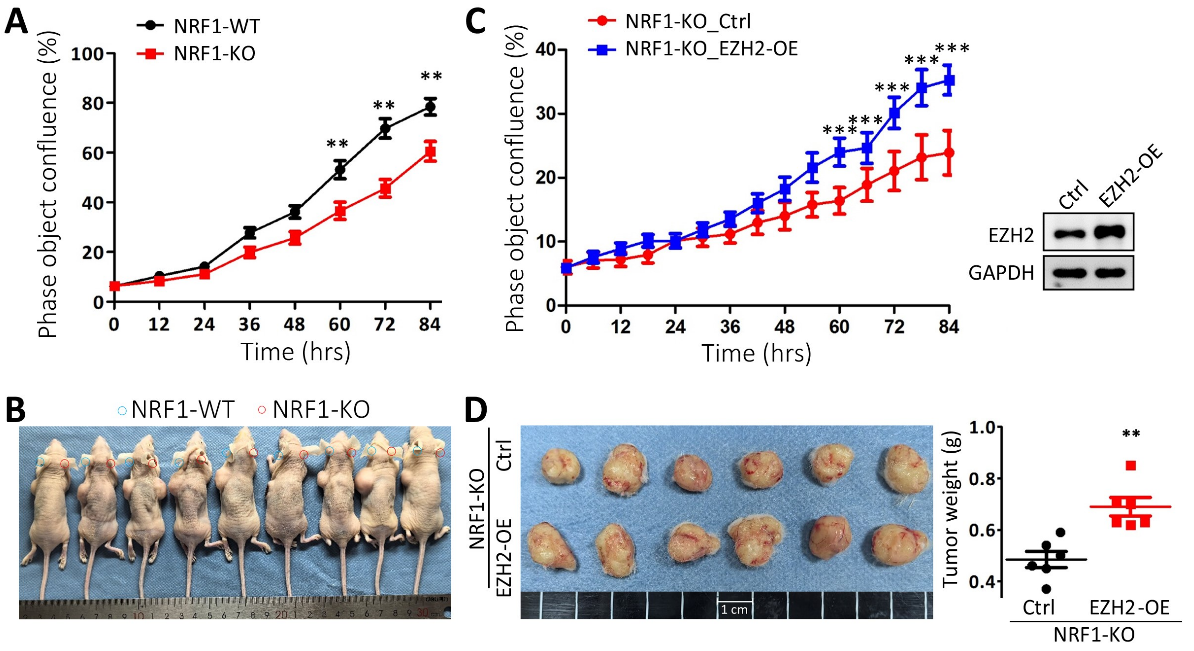


**Figure S5. Effect of *NRF1* knockout and restoration of *EZH2* expression on the proliferation of HCT116 cells.** (**A**) Effect of *NRF1* knockout on HCT116 cell proliferation, as determined by IncuCyte (n=6). (**B**) Effect of *NRF1* knockout on the formation of tumors derived from HCT116 cells in nude mice (n=9). (**C**) Effect of the stable restoration of *EZH2* expression on NRF1-KO HCT116 cell proliferation, as determined by IncuCyte (n=6). Western blot images for EZH2 abundance are inserted on the right. (**D**) Comparison of the weight and size of tumors (n=6) derived from NRF1-KO HCT116 cells with and without the stable restoration of *EZH2* expression. All the data are presented as the means ± SDs. Statistical analysis was performed via two-tailed Student's t test. **P < 0.01, ***P < 0.001

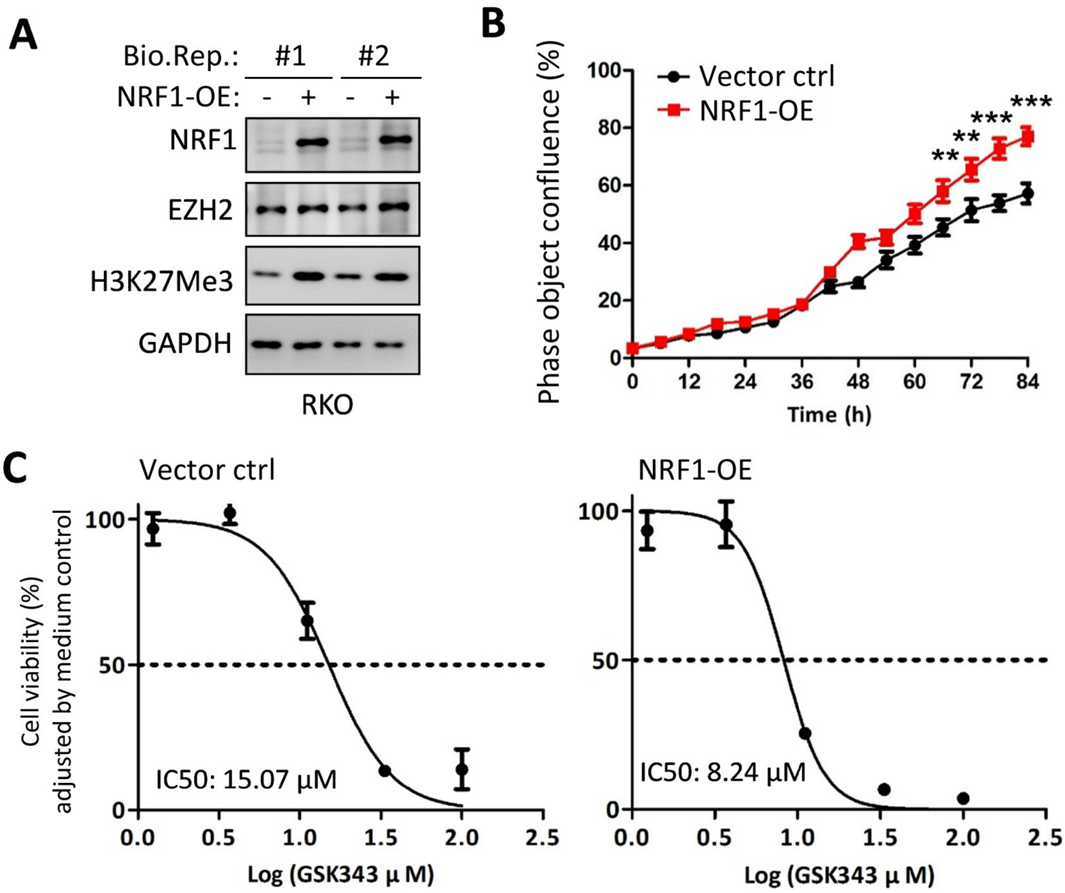


**Figure S6. Effect of NRF1 overexpression (NRF1-OE) on the proliferation of RKO cells and their sensitivity to the EZH2i GSK343.** (**A**) Western blotting images for monitoring alterations in EZH2 and H3K27Me3 levels; (**B**) Proliferation curves of RKO cells with and without stable NRF1-OE, as determined by IncuCyte analysis; All data are presented as the means ± SDs. Statistical analysis was performed via two-tailed Student's t test. **P < 0.01; ***P < 0.001; (**C**) Cell viability curves for determination GSK343 IC50.

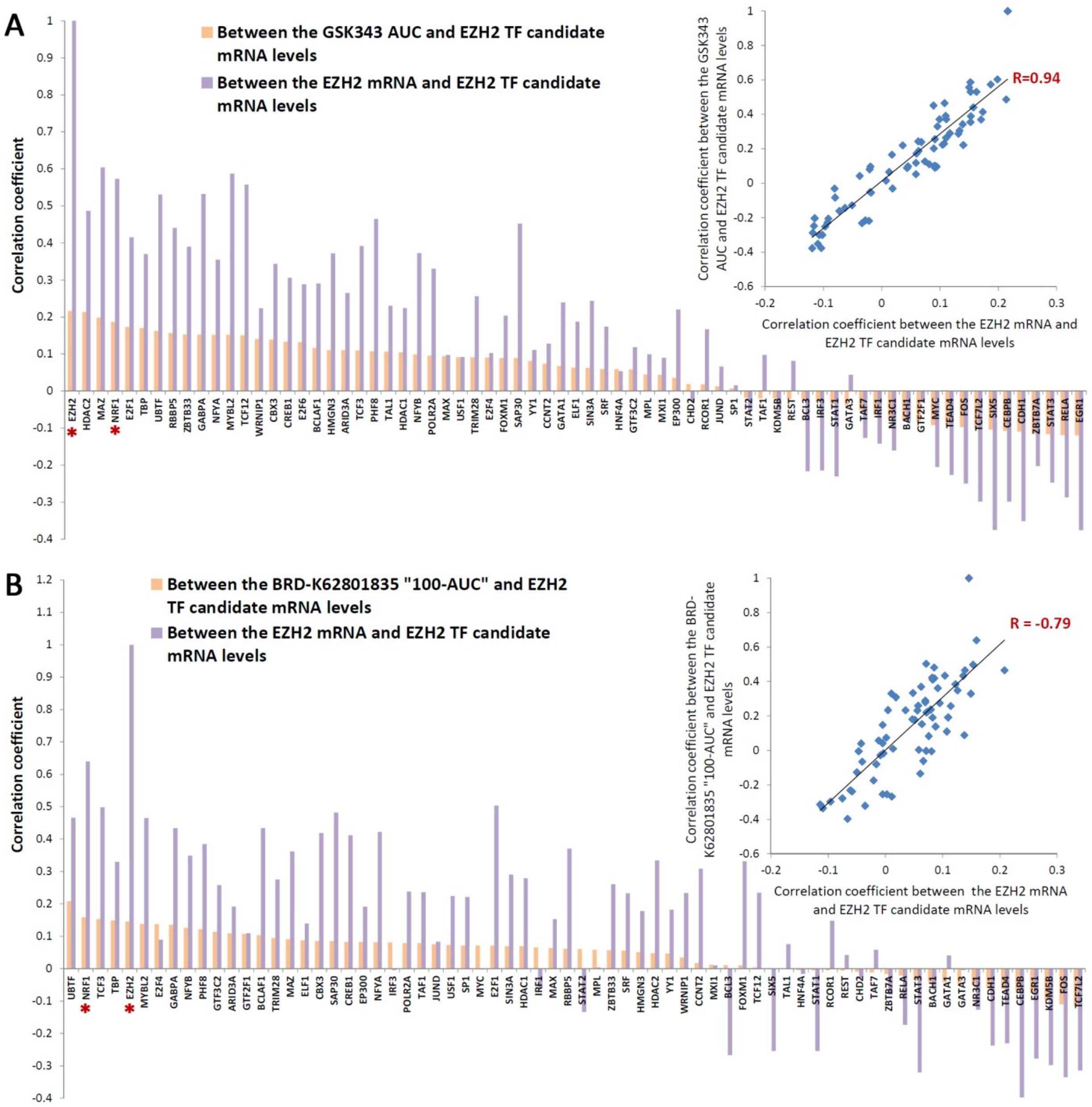


**Figure S7. Correlation coefficients between the mRNA levels of these TF candidates (N=71) and *EZH2* (pink lines) or the AUC of EZH2i (yellow lines).** (**A**) The AUC of EZH2i GSK343, according to GDSC2 data (36); (**B**) The 100-AUC of BRD-K62801835 according to CPRTv2 data (37). The chart for the correlation between the TF-AUC and TF-EZH2 correlation coefficients is inserted within the left. Red stars, EZH2 itself and NRF1. Note: AUC in the CPRTv2.1 data is the area under the percent-viability curve. To make AUC in the CPRTv2.1 data comparable with AUC in the GDSC2 data, we converted CPRTv2.1 AUC as "100-AUC".

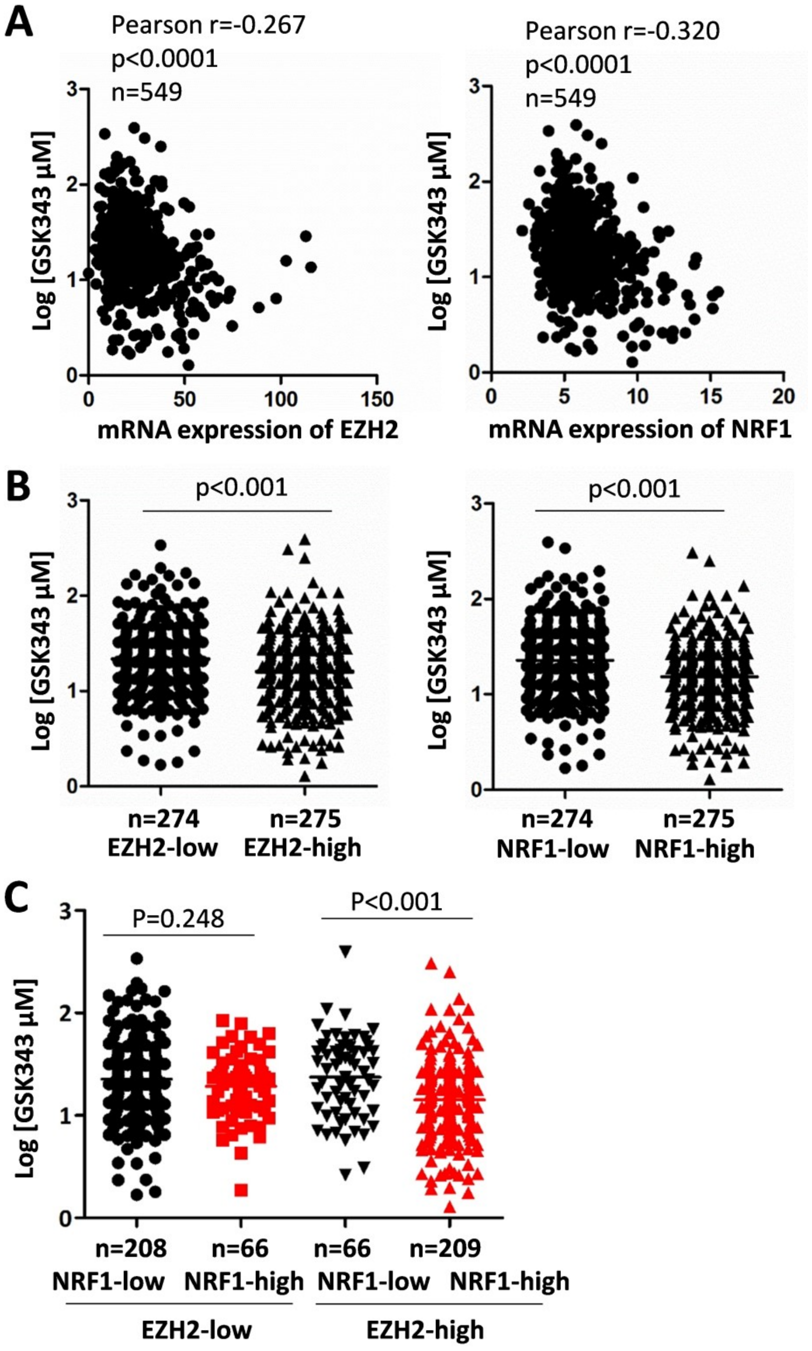


**Figure S8. Relationship between the mRNA level of EZH2 or NRF1 and the IC50 of GSK343.** (**A**) The correlation between the mRNA level of *NRF1* or *EZH2* and the IC50 of the GSK343 inhibitor was analyzed in 549 cancer cell lines from the CCLE datasets (30) and GDSC2 datasets (36). (**B**) The IC50 of GSK343 in cells with high or low *EZH2* or *NRF1* expression. (**C**) The IC50 of GSK343 in NRF1-high cell lines and NRF1-low cell lines with high or low EZH2 expression.

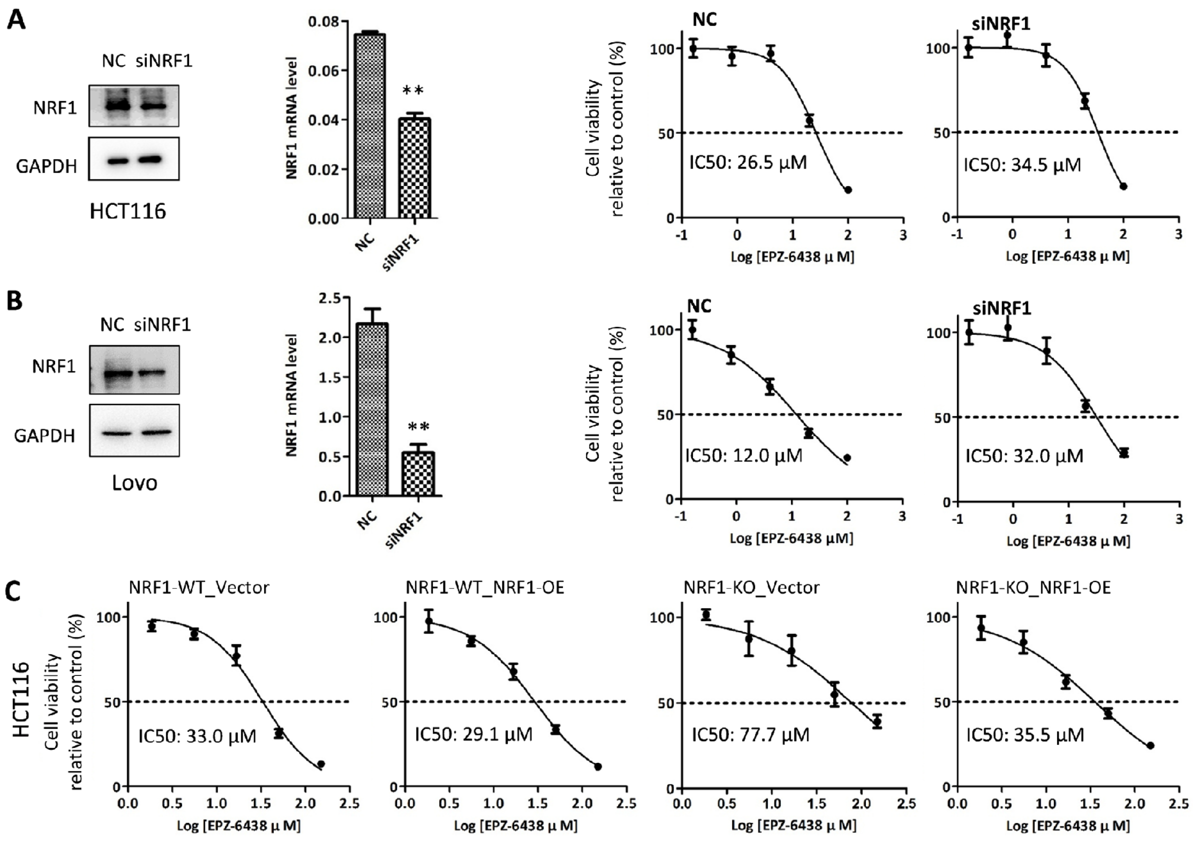


**Figure S9. Effect of changes in *NRF1* expression on the dose–response curve and viability of colon cancer cells in response to tazemetostat (EPZ-6438).** (**A** and **B**) Effect of siNRF1 on the sensitivity of HCT116 and LoVo cells to EPZ6438 treatment. The status of *NRF1* knockdown was monitored by Western blotting and qRT–PCR. (**C**) Effects of *NRF1* knockout and restoration of *NRF1* expression on the sensitivity of HCT116 cells to EPZ6438. The status of *NRF1* restoration was monitored by Western blotting (Figure 5C). EPZ6438-IC50 values are labeled. All the data are presented as the means ± SDs. Statistical analysis was performed via two-tailed Student's t test. **P < 0.01

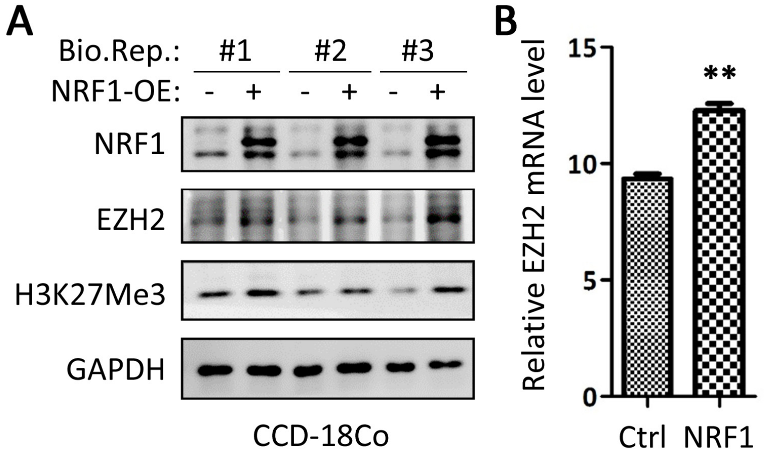


**Figure S10. Effect of NRF1 overexpression on EZH2 expression and its methyltransferase activity for histone 3 lysine 27 (H3K27) in normal human colon CCD-18Co fibroblasts.** (**A**) Western blotting Images; (**B**) The results of RT-qPCR. All data are presented as the means ± SDs. Statistical analysis was performed via two-tailed Student's t test. **P < 0.01

**Supplemental file**

Raw images for Western blot analyses.
