# Supplementary material for: NRF1 predominantly causes EZH2 overexpression in cancer cells
Source: Cell Death Dis. 2026 May 16;17(1):625. doi: 10.1038/s41419-026-08861-4 (PMC13346543; doi:10.1038/s41419-026-08861-4)
Supplement: Supplementary file 2 — Raw images for Western blotting [file 41419_2026_8861_MOESM2_ESM.pdf]

Figure 1A

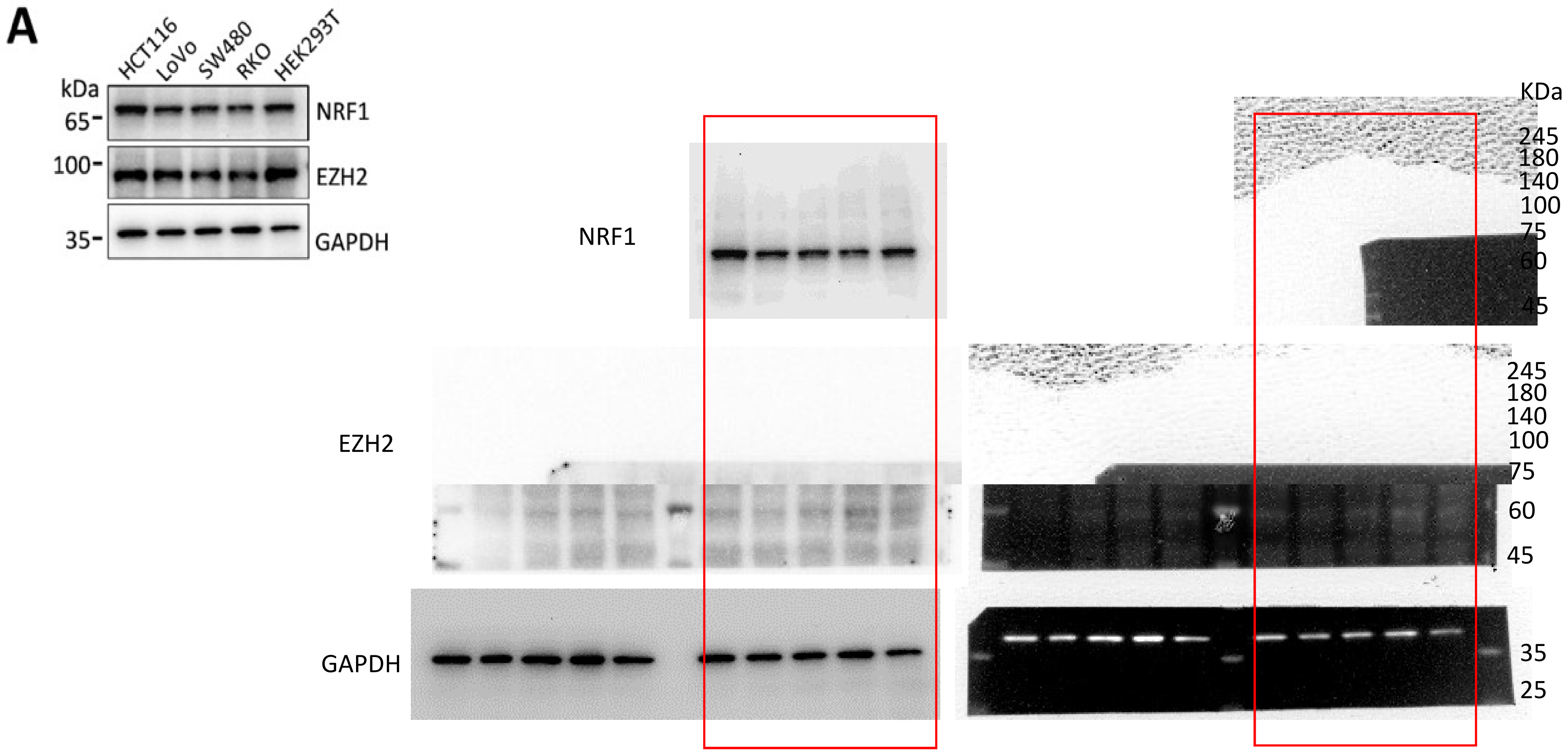
A
HCT116
LoVo
SW480
RKO
HEK293T
kDa
65
100
35
NRF1
EZH2
GAPDH
NRF1
EZH2
GAPDH
KDa
245
180
140
100
75
60
45
245
180
140
100
75
60
45
35
25

Figure 1B

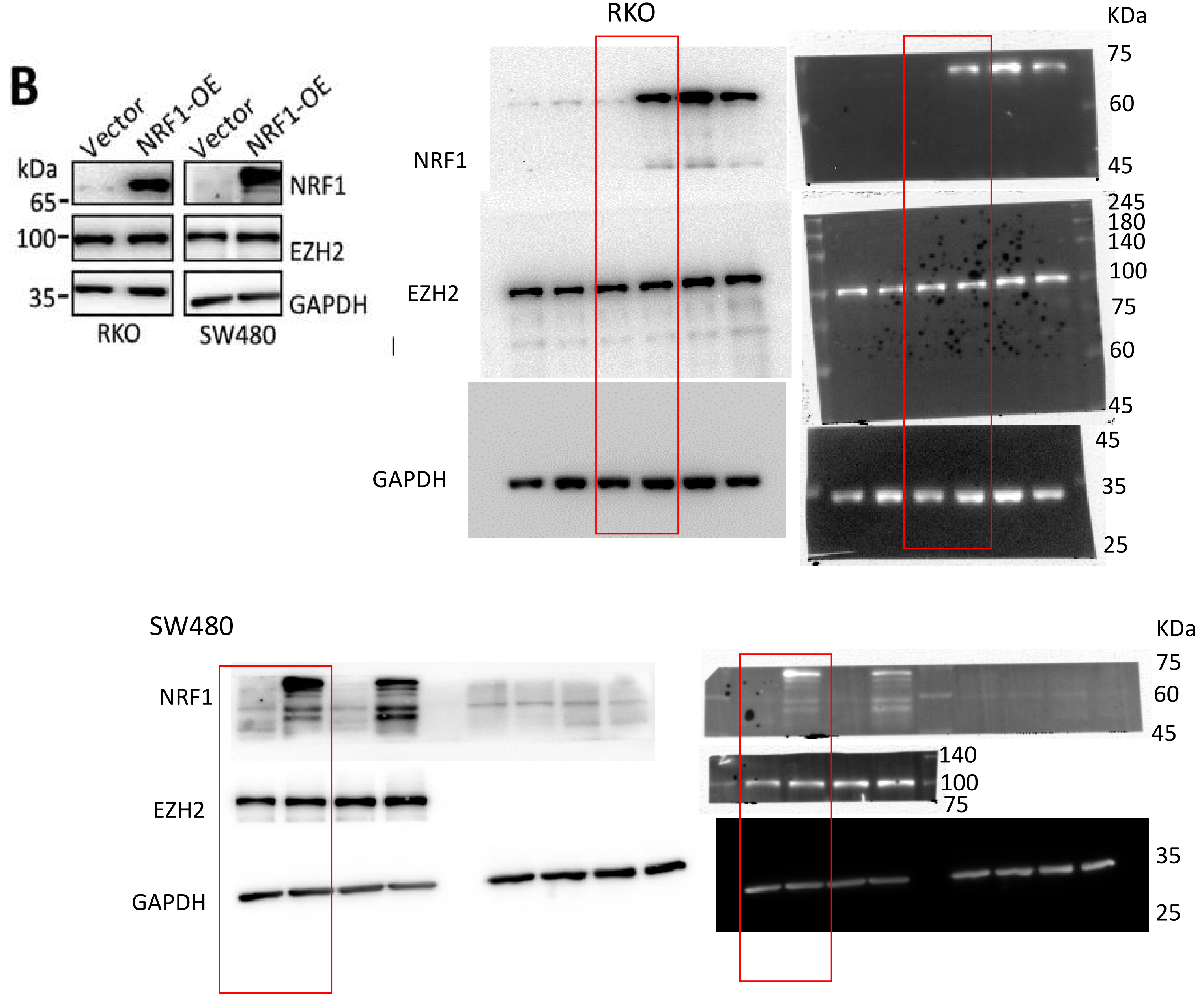
B
Vector
NRF1-OE
Vector
NRF1-OE
kDa
65
100
35
NRF1
EZH2
GAPDH
RKO
SW480
RKO
NRF1
EZH2
GAPDH
KDa
75
60
45
245
180
140
100
75
60
45
45
35
25
SW480
NRF1
EZH2
GAPDH
KDa
75
60
45
140
100
75
35
25

Figure 1C

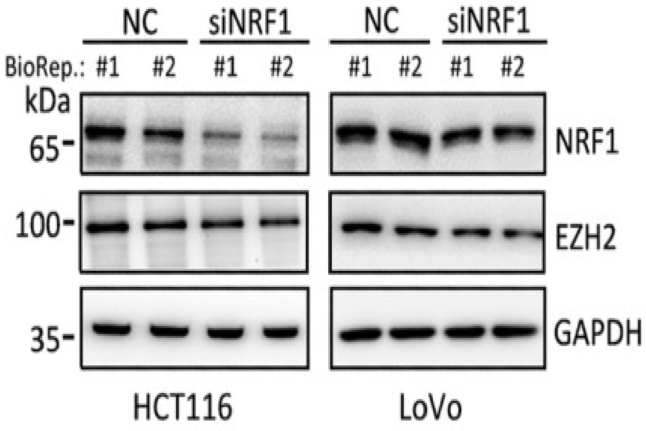
NC
siNRF1
NC
siNRF1
BioRep.: #1 #2 #1 #2 #1 #2 #1 #2
kDa
65
100
35
NRF1
EZH2
GAPDH
HCT116
LoVo

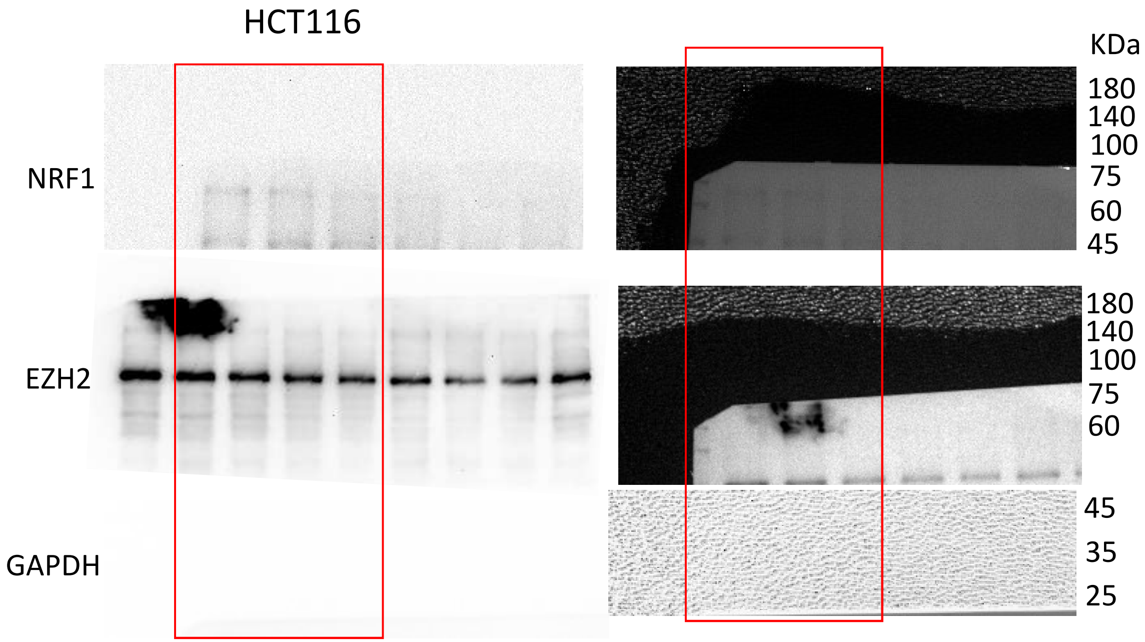
HCT116
NRF1
EZH2
GAPDH
KDa
180
140
100
75
60
45
35
25

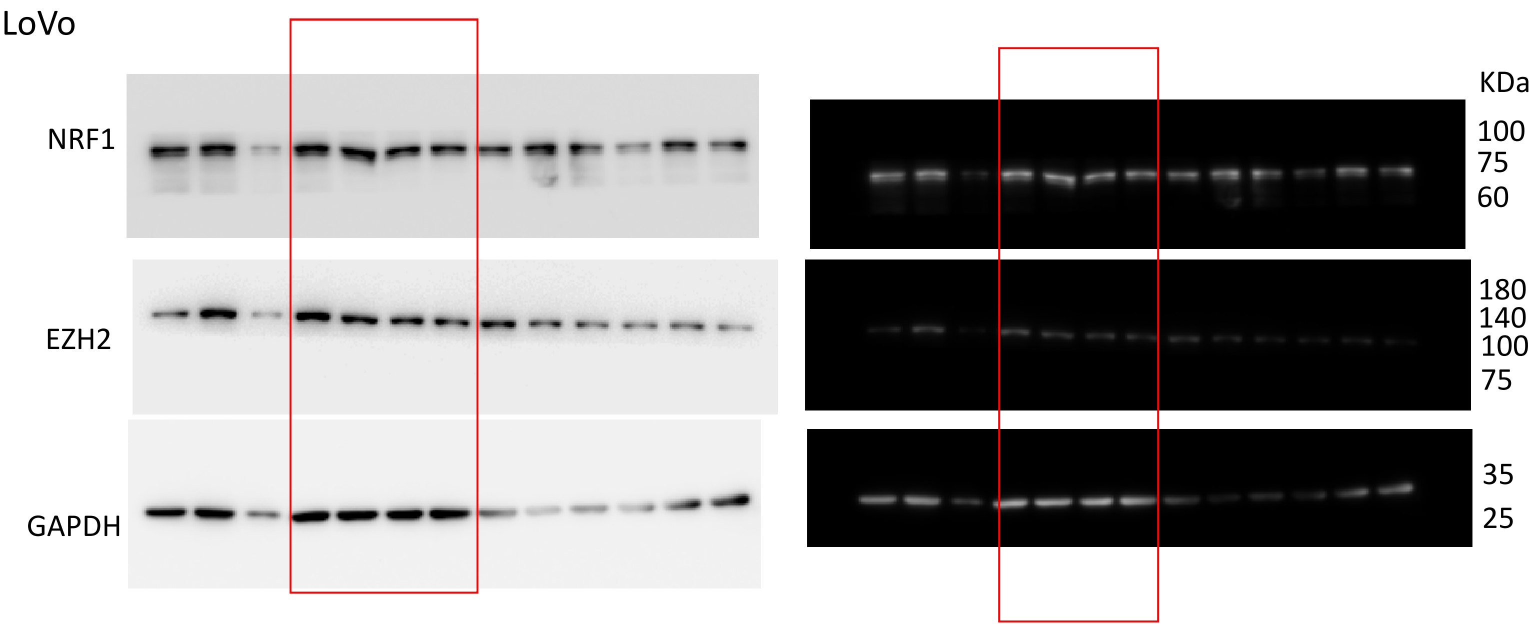
LoVo
NRF1
EZH2
GAPDH
KDa
100
75
60
180
140
100
75
35
25

Figure 1D

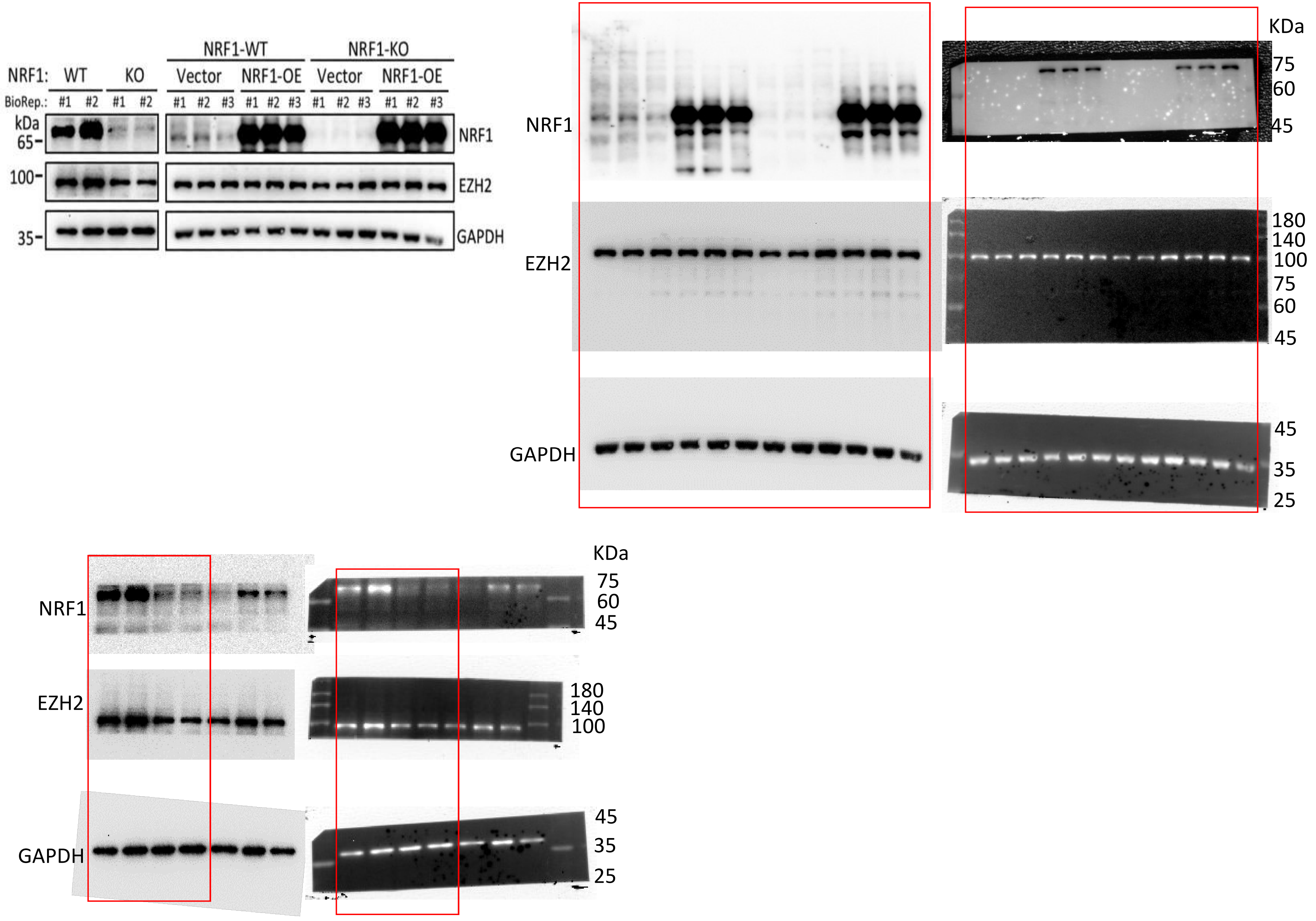
NRF1-WT
NRF1-KO
NRF1: WT KO Vector NRF1-OE Vector NRF1-OE
BioRep.: #1 #2 #1 #2 #1 #2 #3 #1 #2 #3 #1 #2 #3 #1 #2 #3
kDa
65
100
35
NRF1
EZH2
GAPDH
KDa
75
60
45
180
140
100
75
60
45
45
35
25
NRF1
EZH2
GAPDH
KDa
75
60
45
180
140
100
45
35
25

Figure 2A

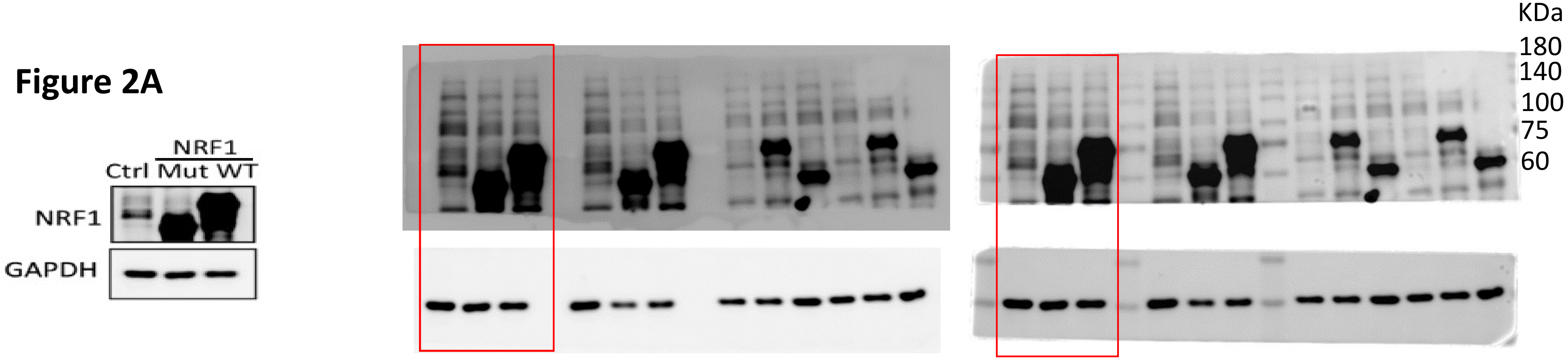

Figure 2C

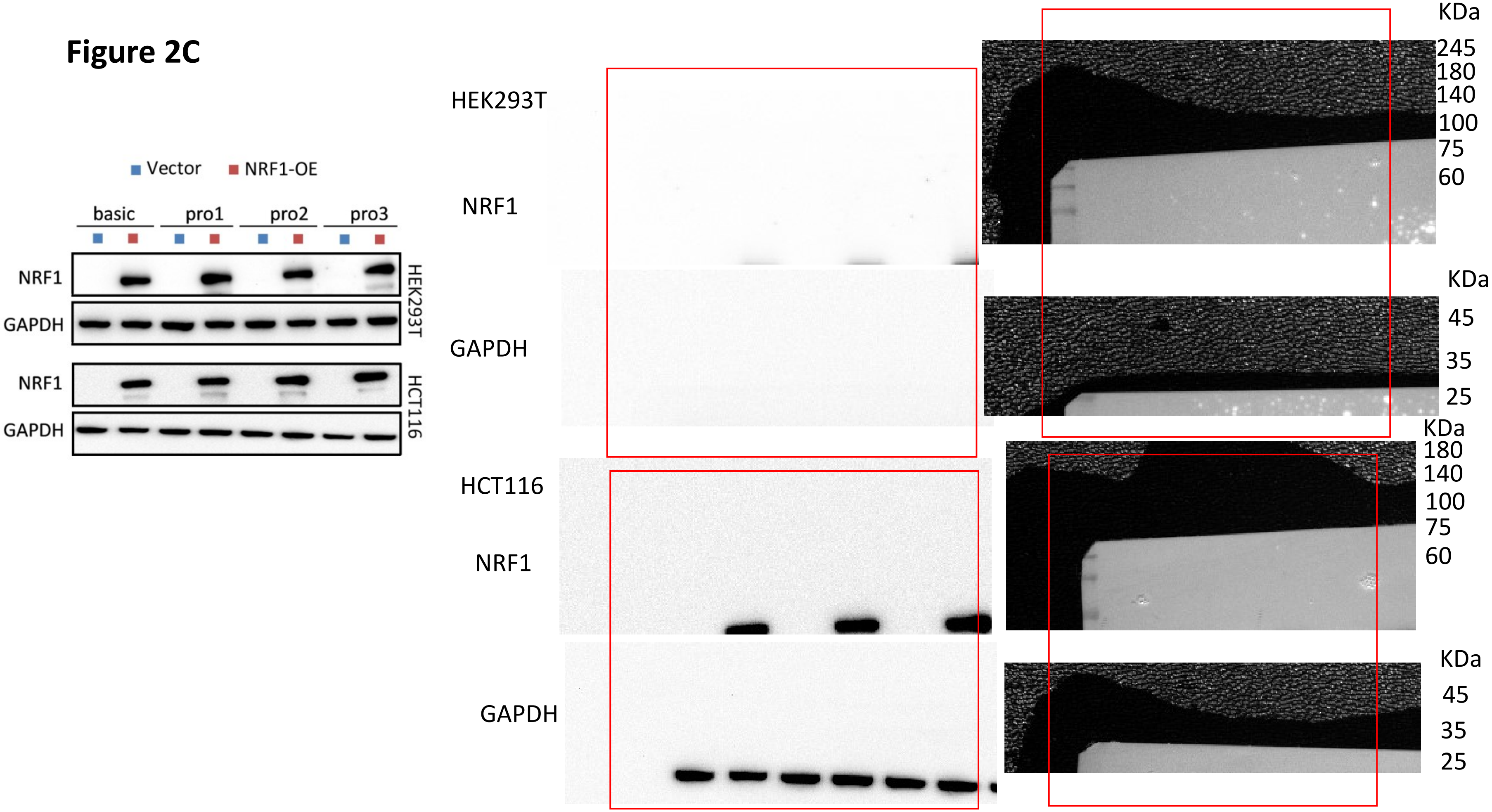

Figure 2D

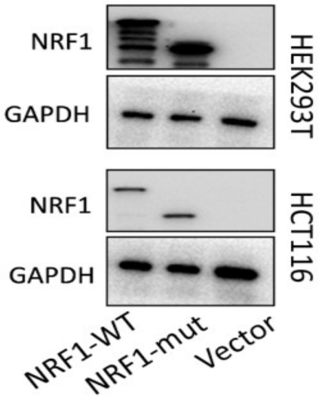
NRF1
GAPDH
NRF1
GAPDH
HEK293T
HCT116
NRF1-WT
NRF1-mut
Vector

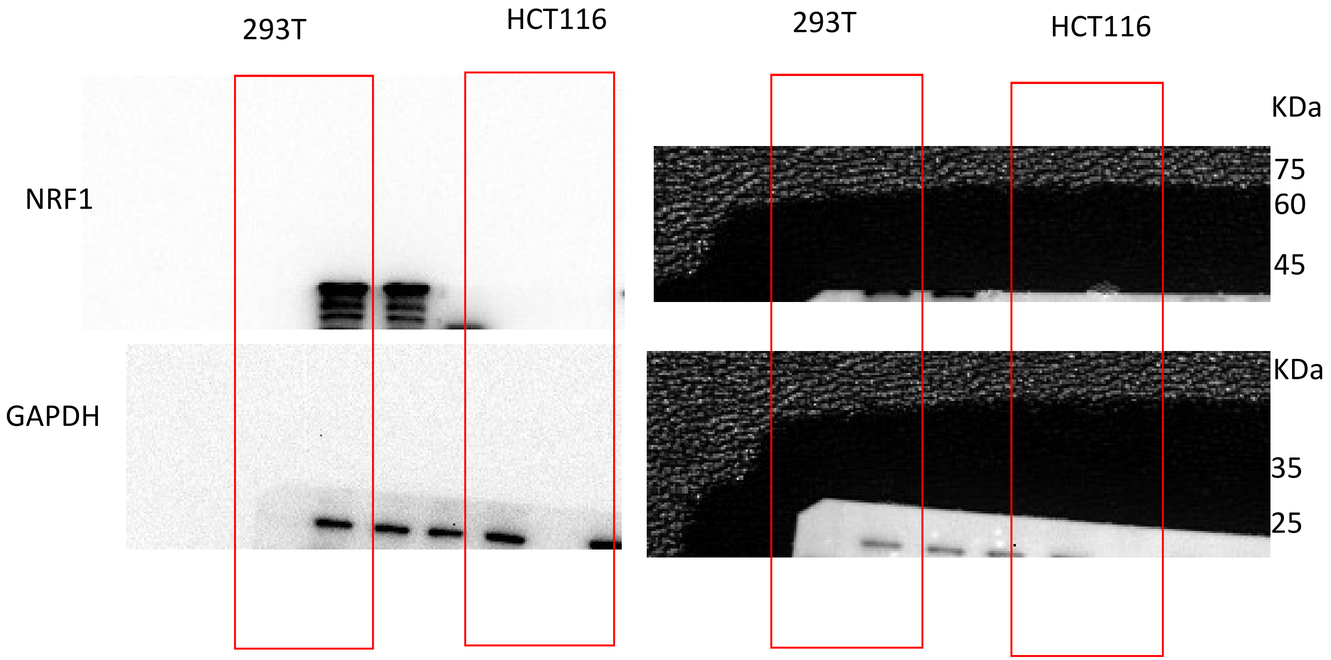
293T
HCT116
293T
HCT116
NRF1
GAPDH
KDa
75
60
45
KDa
35
25

Figure 3B-3C

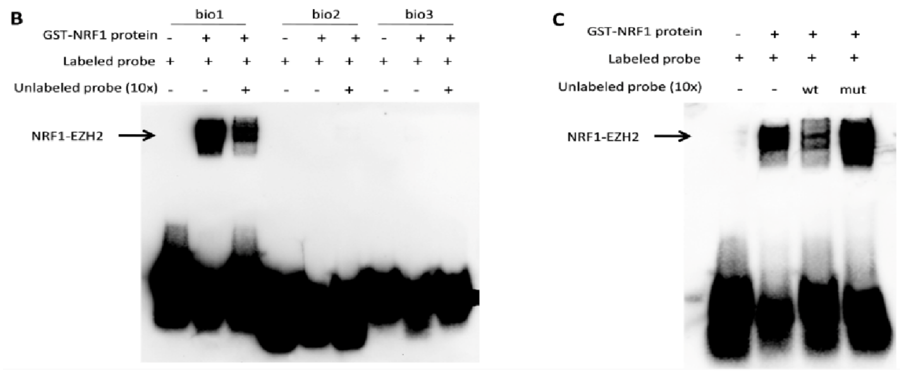
B
bio1
bio2
bio3
GST-NRF1 protein - + + - + + - + +
Labeled probe + + + + + + + + +
Unlabeled probe (10x) - - + - - + - - +
NRF1-EZH2
C
GST-NRF1 protein - + + +
Labeled probe + + + +
Unlabeled probe (10x) - - wt mut
NRF1-EZH2

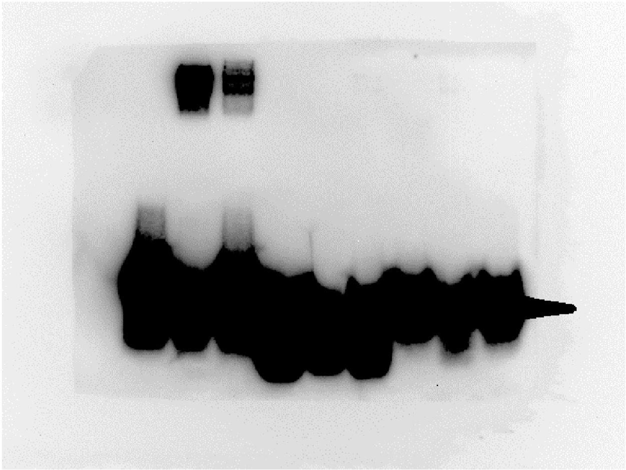

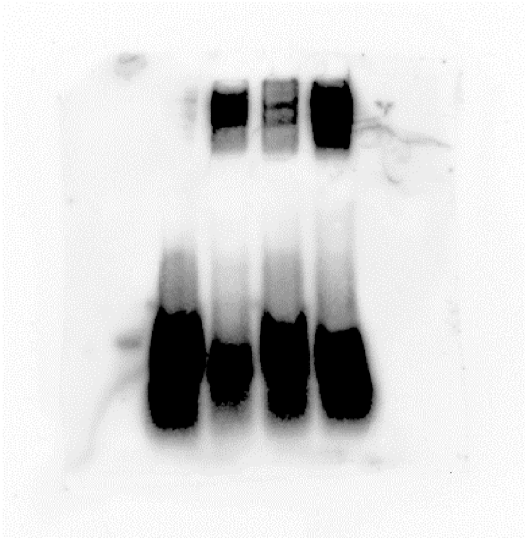

Figure 5A-5B

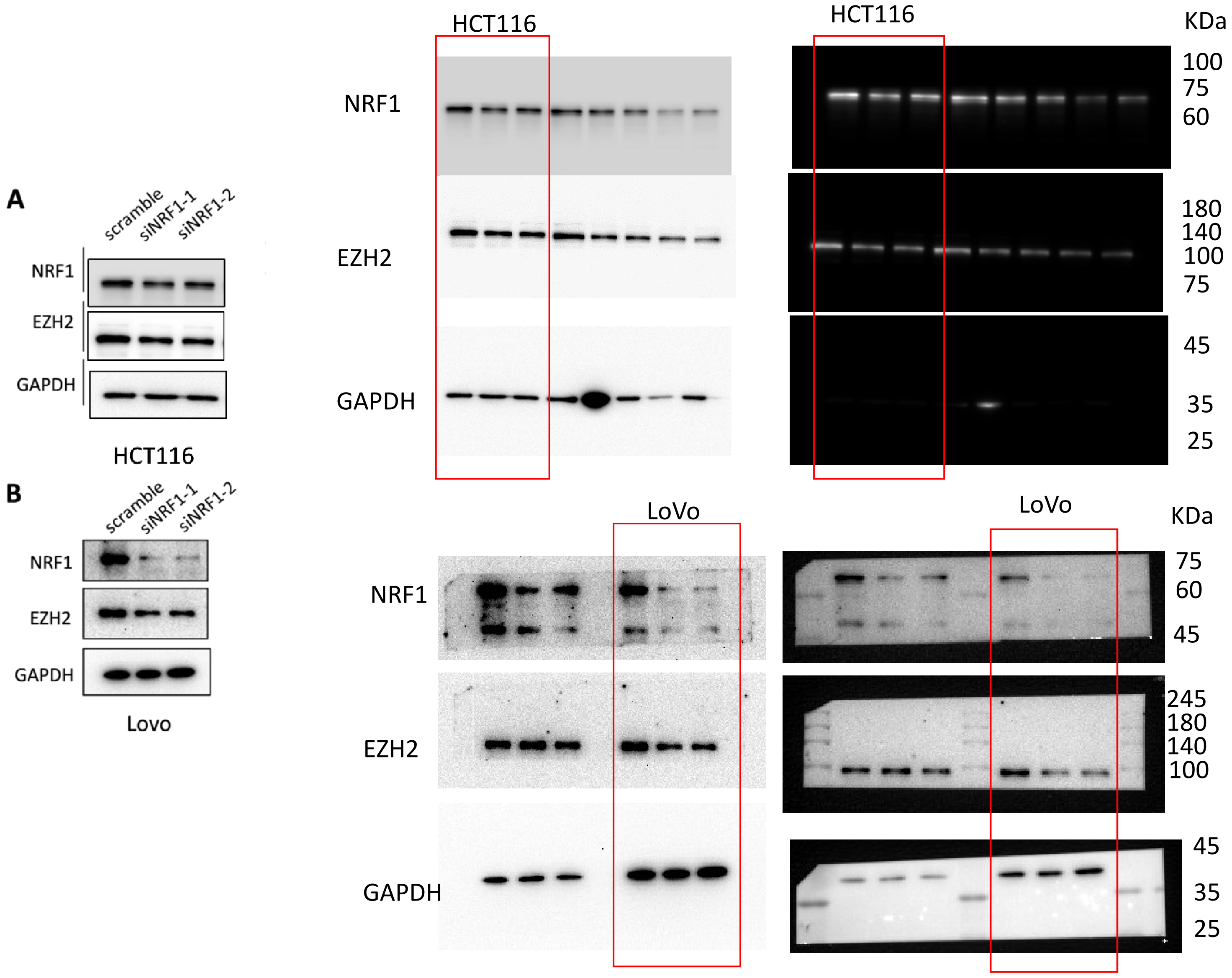

A
scramble
siNRF1-1
siNRF1-2
NRF1
EZH2
GAPDH
HCT116
B
scramble
siNRF1-1
siNRF1-2
NRF1
EZH2
GAPDH
Lovo
HCT116
NRF1
EZH2
GAPDH
HCT116
KDa
100
75
60
180
140
100
75
45
35
25
LoVo
NRF1
EZH2
GAPDH
LoVo
KDa
75
60
45
245
180
140
100
45
35
25

Figure 5C

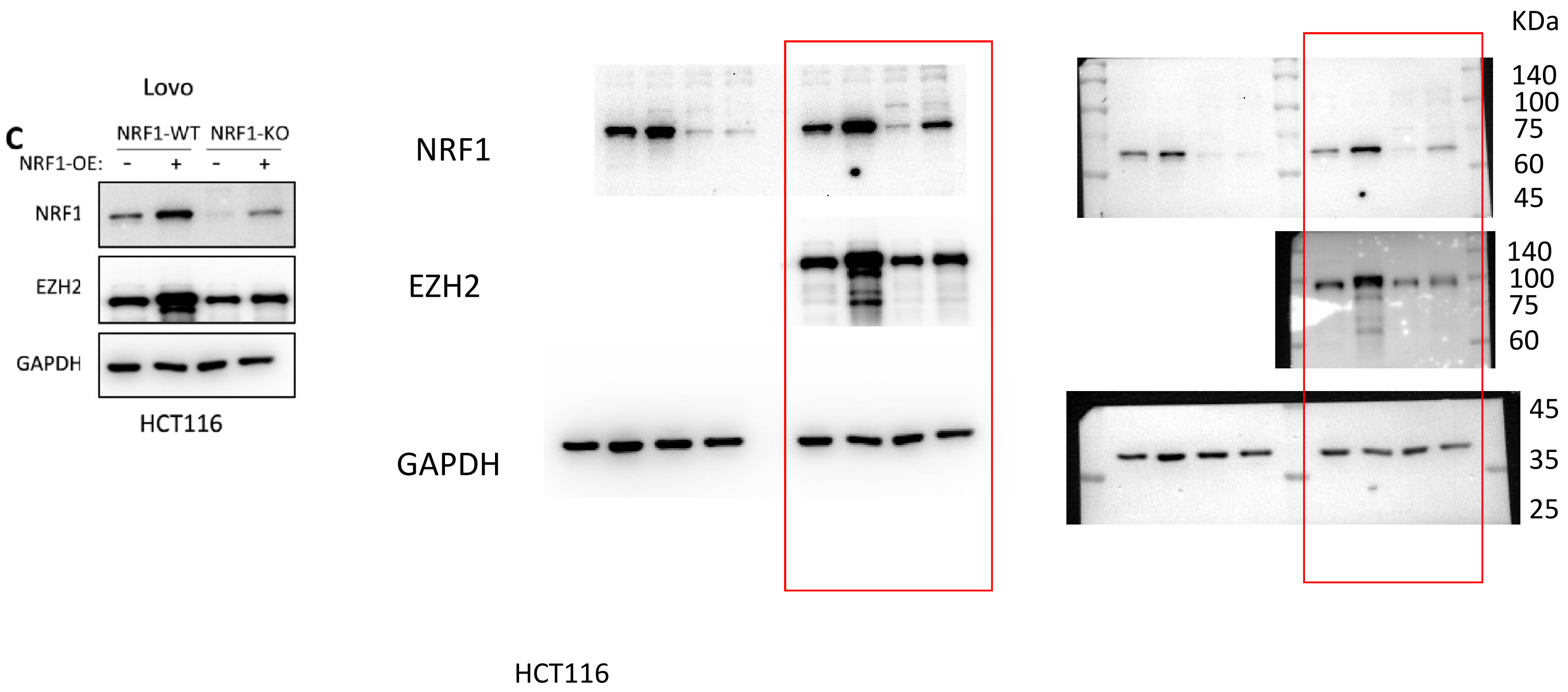

Lovo
C
NRF1-WT
NRF1-KO
NRF1-OE: - + - +
NRF1
EZH2
GAPDH
HCT116
NRF1
EZH2
GAPDH
KDa
140
100
75
60
45
140
100
75
60
45
35
25
HCT116

Figure 6B-6C

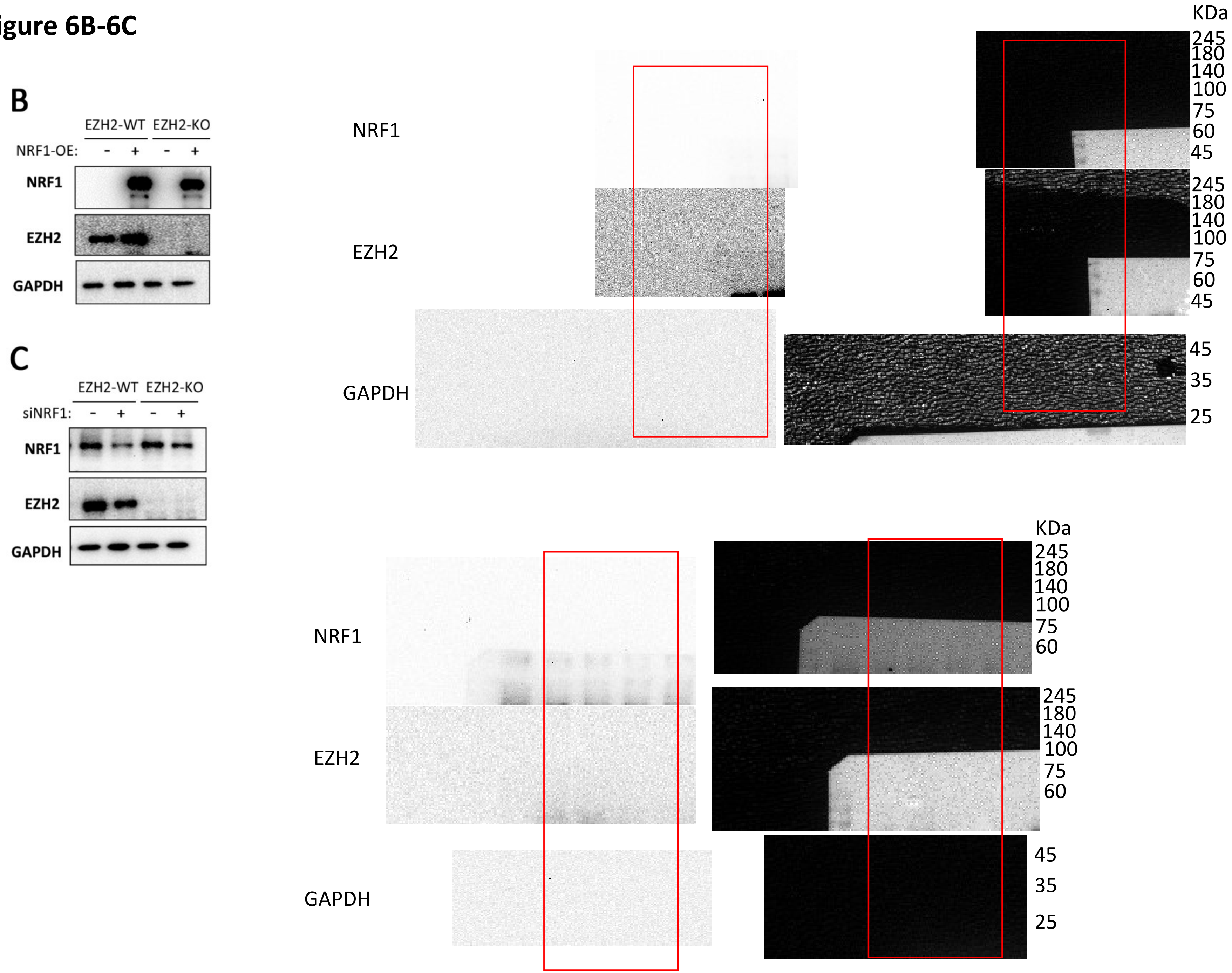
B
EZH2-WT
EZH2-KO
NRF1-OE:
-
+
-
+
NRF1
EZH2
GAPDH
C
EZH2-WT
EZH2-KO
siNRF1:
-
+
-
+
NRF1
EZH2
GAPDH
NRF1
EZH2
GAPDH
KDa
245
180
140
100
75
60
45
245
180
140
100
75
60
45
45
35
25
NRF1
EZH2
GAPDH
KDa
245
180
140
100
75
60
245
180
140
100
75
60
45
35
25

Figure 7E

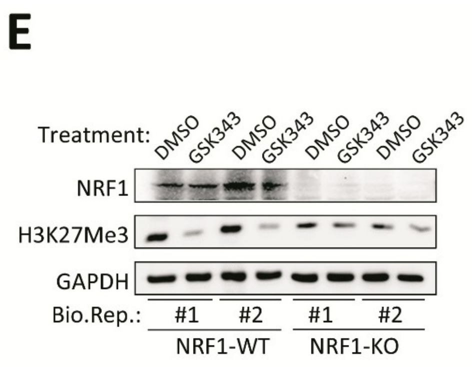
E
Treatment:
DMSO
GSK343
DMSO
GSK343
DMSO
GSK343
DMSO
GSK343
NRF1
H3K27Me3
GAPDH
Bio.Rep.:
#1
#2
#1
#2
NRF1-WT
NRF1-KO

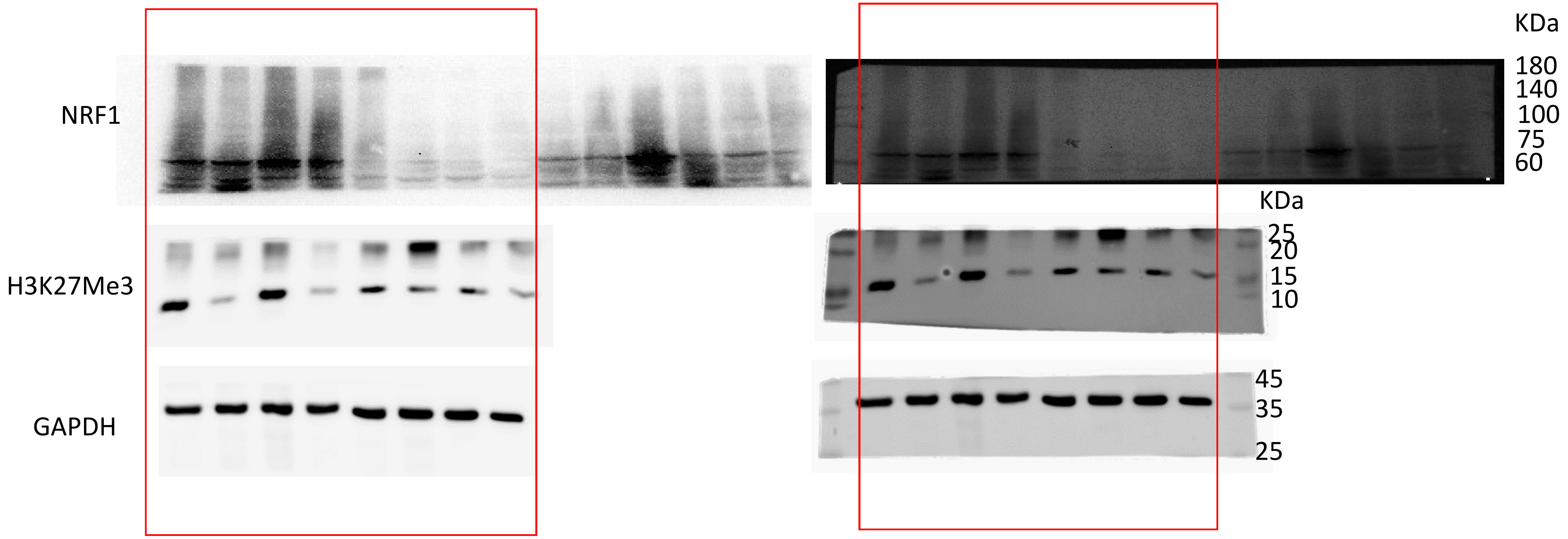
NRF1
H3K27Me3
GAPDH
KDa
180
140
100
75
60
KDa
25
20
15
10
45
35
25

Figure S1A/C

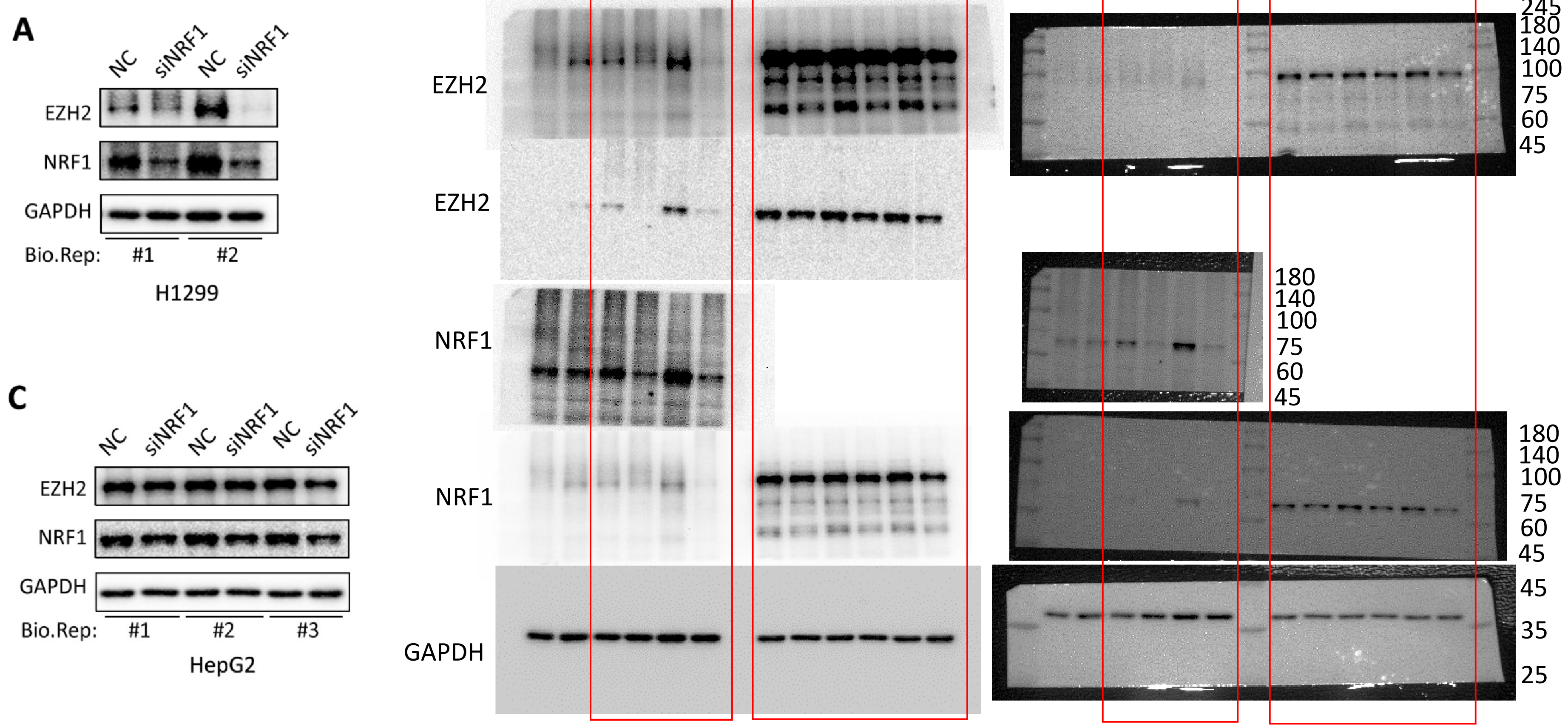
A
NC
siNRF1
NC
siNRF1
EZH2
NRF1
GAPDH
Bio.Rep:
#1
#2
H1299
C
NC
siNRF1
NC
siNRF1
NC
siNRF1
EZH2
NRF1
GAPDH
Bio.Rep:
#1
#2
#3
HepG2
H1299
HepG2
EZH2
EZH2
NRF1
NRF1
GAPDH
H1299
HepG2
KDa
245
180
140
100
75
60
45
180
140
100
75
60
45
180
140
100
75
60
45
45
35
25

Figure S1B

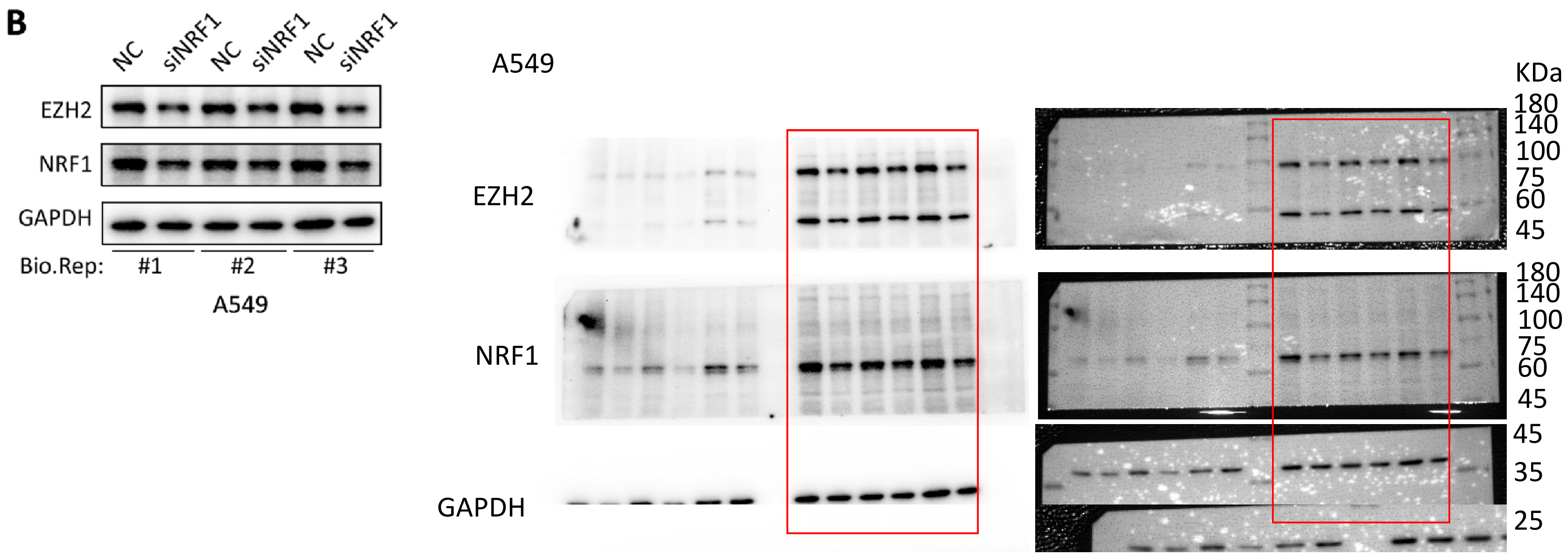
B
NC
siNRF1
NC
siNRF1
NC
siNRF1
EZH2
NRF1
GAPDH
Bio.Rep:
#1
#2
#3
A549
A549
EZH2
NRF1
GAPDH
KDa
180
140
100
75
60
45
180
140
100
75
60
45
45
35
25

Figure S5C

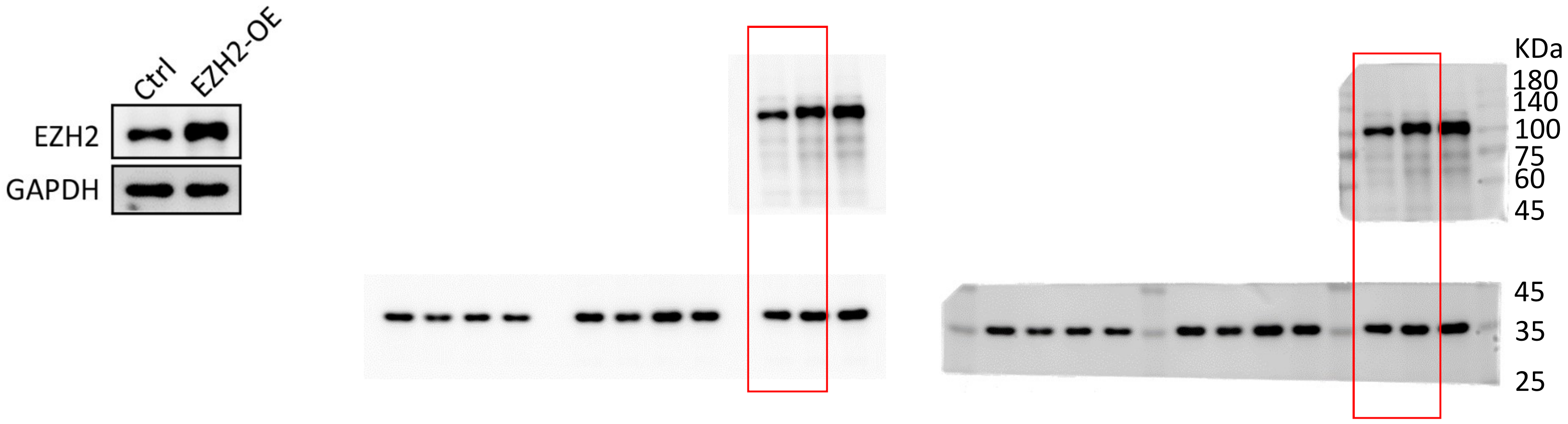

Figure S6A

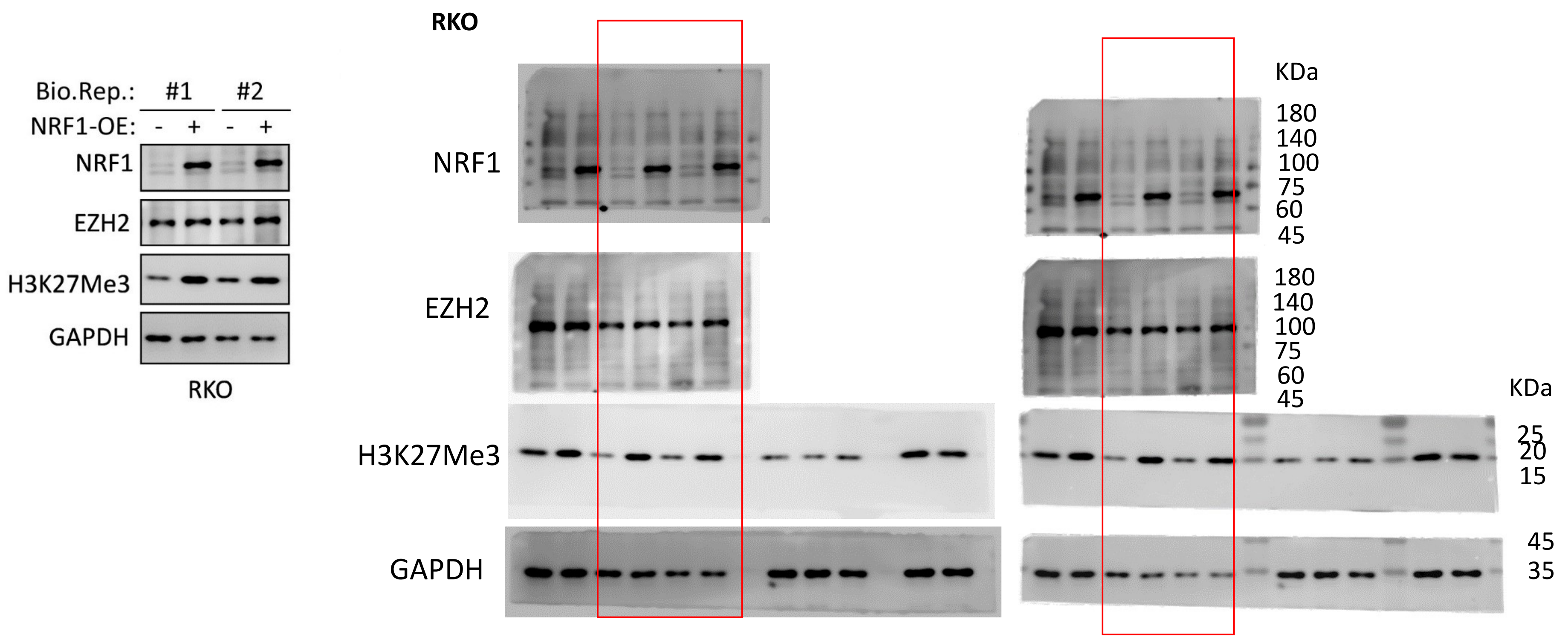

Bio.Rep.: #1 #2
NRF1-OE: - + - +
NRF1
EZH2
H3K27Me3
GAPDH
RKO
RKO
NRF1
EZH2
H3K27Me3
GAPDH
KDa
180
140
100
75
60
45
180
140
100
75
60
45
KDa
25
20
15
45
35

Figure S9A-9B

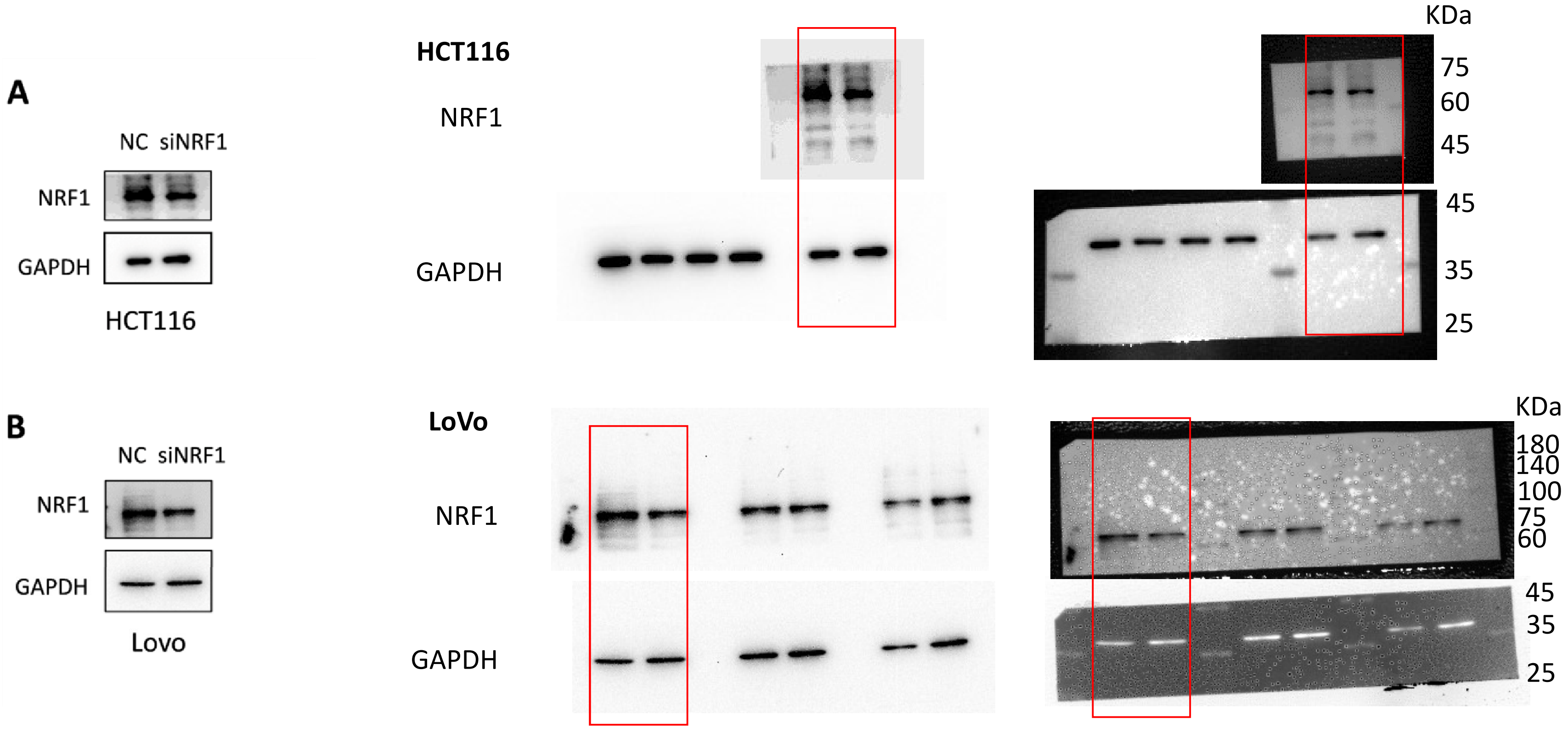

Figure S10A

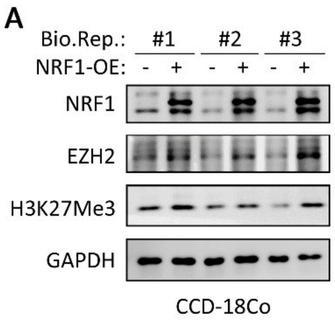
A
Bio.Rep.: #1 #2 #3
NRF1-OE: - + - + - +
NRF1
EZH2
H3K27Me3
GAPDH
CCD-18Co

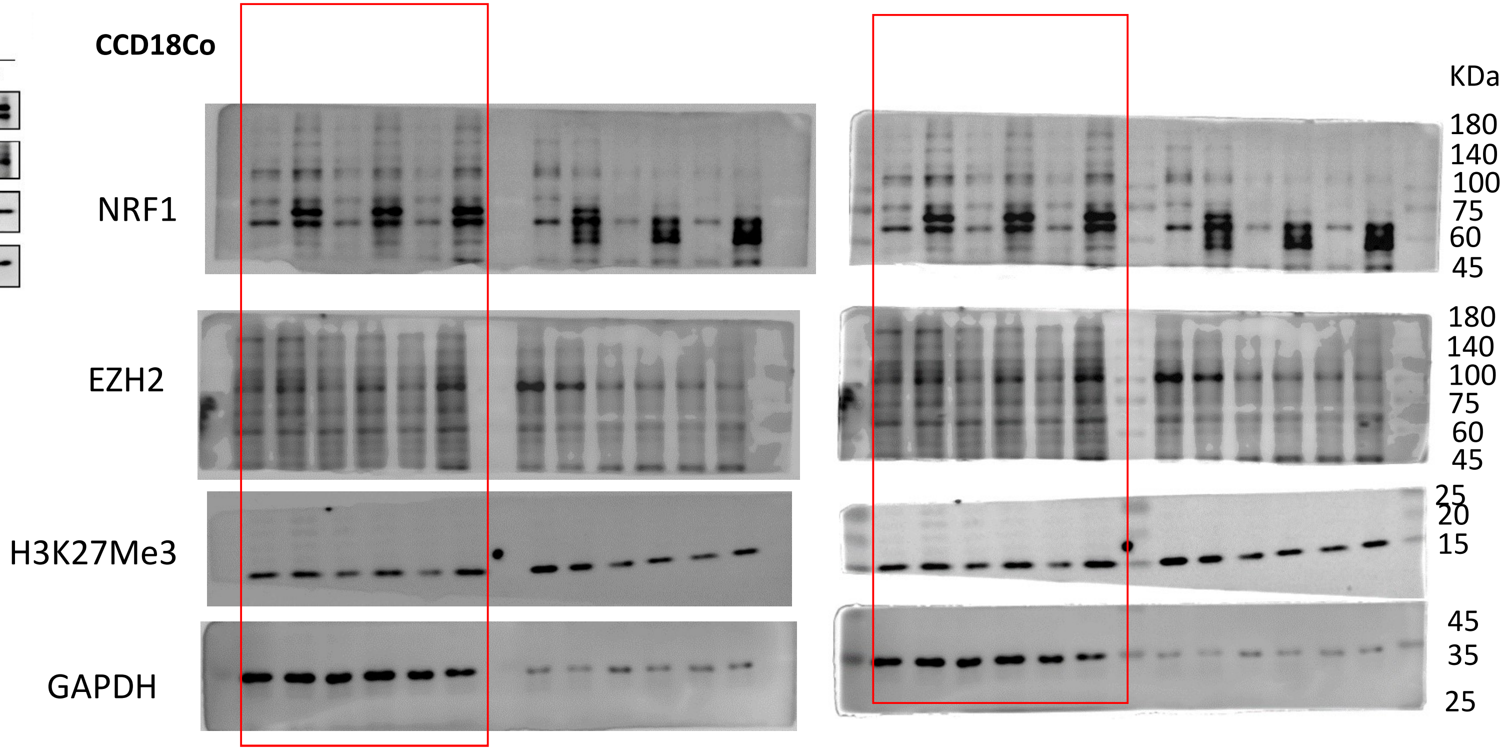
CCD18Co
NRF1
EZH2
H3K27Me3
GAPDH
KDa
180
140
100
75
60
45
180
140
100
75
60
45
25
20
15
45
35
25

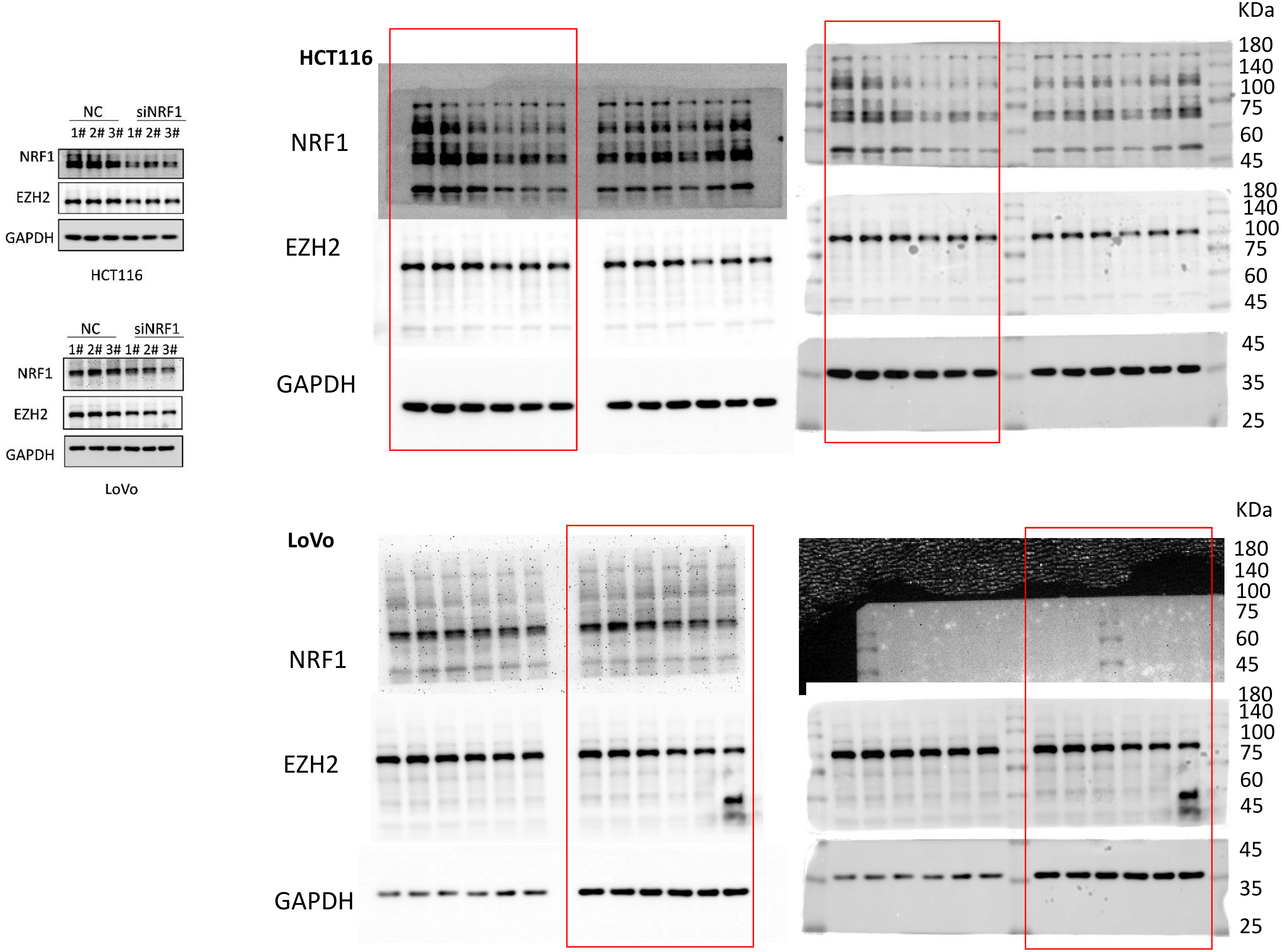
NC
siNRF1
1# 2# 3# 1# 2# 3#
NRF1
EZH2
GAPDH
HCT116
NC
siNRF1
1# 2# 3# 1# 2# 3#
NRF1
EZH2
GAPDH
LoVo
HCT116
NRF1
EZH2
GAPDH
KDa
180
140
100
75
60
45
180
140
100
75
60
45
45
35
25
LoVo
NRF1
EZH2
GAPDH
KDa
180
140
100
75
60
45
180
140
100
75
60
45
45
35
25
